# Complex plasmon-exciton dynamics revealed through quantum dot light emission in a nanocavity

**DOI:** 10.1038/s41467-021-21539-z

**Published:** 2021-02-26

**Authors:** Satyendra Nath Gupta, Ora Bitton, Tomas Neuman, Ruben Esteban, Lev Chuntonov, Javier Aizpurua, Gilad Haran

**Affiliations:** 1 Department of Chemical and Biological Physics, Weizmann Institute of Science, Rehovot, Israel; 2 Department of Chemical Research Support, Weizmann Institute of Science, Rehovot, Israel; 3 Materials Physics Center CSIC-UPV/EHU, Paseo Manuel de Lardizabal 5, Donostia-San Sebastián, Spain; 4 Donostia International Physics Center DIPC, Paseo Manuel de Lardizabal 4, Donostia-San Sebastián, Spain; 5 Schulich Faculty of Chemistry, Technion-Israel Institute of Technology, Haifa, Israel

**Keywords:** Nanophotonics and plasmonics, Quantum optics, Chemical physics

## Abstract

Plasmonic cavities can confine electromagnetic radiation to deep sub-wavelength regimes. This facilitates strong coupling phenomena to be observed at the limit of individual quantum emitters. Here, we report an extensive set of measurements of plasmonic cavities hosting one to a few semiconductor quantum dots. Scattering spectra show Rabi splitting, demonstrating that these devices are close to the strong coupling regime. Using Hanbury Brown and Twiss interferometry, we observe non-classical emission, allowing us to directly determine the number of emitters in each device. Surprising features in photoluminescence spectra point to the contribution of multiple excited states. Using model simulations based on an extended Jaynes-Cummings Hamiltonian, we find that the involvement of a dark state of the quantum dots explains the experimental findings. The coupling of quantum emitters to plasmonic cavities thus exposes complex relaxation pathways and emerges as an unconventional means to control dynamics of quantum states.

## Abstract (web summary)

Strong coupling of quantum emitters to plasmonic cavities allows exploring rich phenomenologies of light-matter interaction. Here, the authors report experiments on single colloidal quantum dots coupled to plasmonic metal nanostructures, revealing complex interactions between bright and dark states.

## Introduction

Manipulating and controlling the interaction of photons with individual quantum emitters has been a major goal of quantum photonics in recent years^1–3^. Such control can be realized by engineering the local photonic environment of the quantum emitter, e.g. by placing it inside an optical cavity^4^. By coupling the excited state(s) of the emitter to the electromagnetic (EM) field of the cavity, one can generate various exotic light-matter coupled states^1,2^, single-photon emission sources^5,6^ and photonic switches^7,8^. In recent years it has been shown that the formation of new hybrid light-matter states (polaritons) within optical cavities can dramatically affect photophysics^9–11^ and chemical reactivity^12,13^.

Plasmonic cavities (PCs) formed by metallic surfaces can tightly confine light to deep sub-wavelength regimes^14,15^. The ability to strongly couple quantum emitters to individual PCs has aroused much excitement in recent years^16–19^. Our lab^20,21^ and others’^22–25^ have demonstrated that such a strong coupling can be realized even in the limit of a single semiconductor nanocrystal (quantum dot, QD) or molecule, and can be observed as vacuum Rabi splitting in light scattering, photoluminescence (PL) or electron energy loss spectra of the coupled systems. Plasmonic cavities with coupled quantum emitters may serve as new testbeds for studies of quantum optical and chemical dynamics under ambient conditions.

In this work, we expose the remarkable modulation of the excited-state dynamics of QDs embedded within PCs through the comparative analysis of an extensive set of light scattering and PL measurements. Using Hanbury Brown and Twiss (HBT) interferometry, we observe non-classical emission from one to three QDs within our devices, and find surprisingly long excited-state relaxation times. We interpret the experimental results using simulations based on an extended Jaynes–Cummings model, which considers the coexistence of bright and dark excited states in the QD with very different coupling properties. We demonstrate the crucial role that the dark state of the QDs plays in the observed dynamics, and thus in shaping of the final PL spectrum.

## Results

### Scattering and Photoluminescence spectra of individual coupled plasmonic devices

**Fig. 1:**
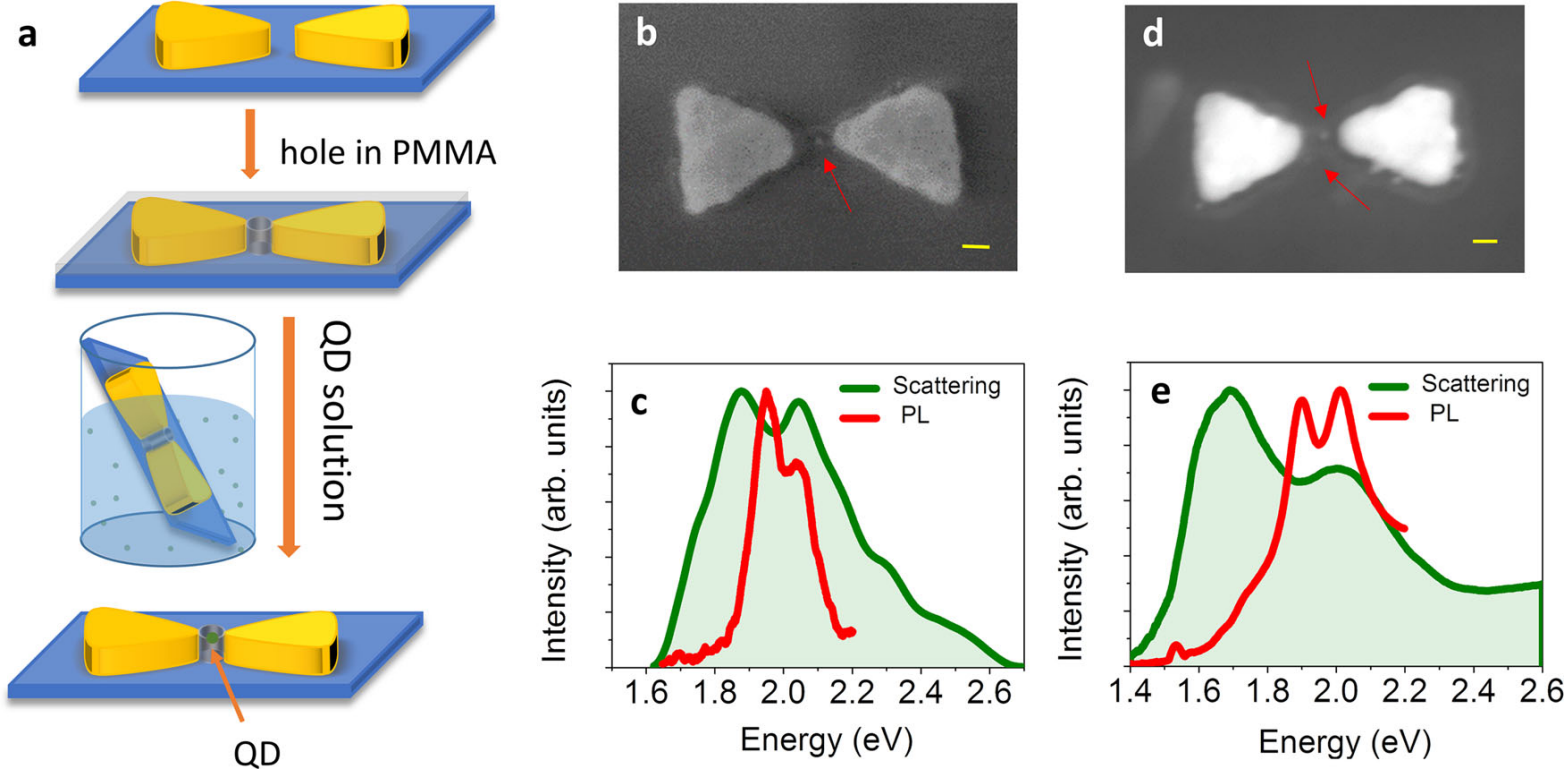
Spectroscopy of plasmonic cavities with QDs. **a** Schematic of the preparative process for trapping QDs within plasmonic bowties. **b**, **d** STEM images of a device with one QD (**b**) and two QDs (**d**). The scale bars represent 20 nm. The red arrows point to the QDs in the bowtie gaps. **c**, **e** Dark-field scattering spectra (green) and PL spectra (red) of the devices in (**b**), (**d**), respectively.

We used electron-beam lithography to fabricate silver bowties on 18 nm SiO_2_ membranes. CdSe/ZnS quantum dots (QDs, obtained from MK Impex Corp. with a size of 6–8 nm) were positioned into the gap region of bowties using interfacial capillary forces (Fig. 1a). Coupling rates in such devices can exceed 100 meV, depending on the position within the cavity^20^. Scattering spectra of individual QD-bowtie hybrids were measured using dark-field (DF) microspectrometry^20,26^, while PL spectra were measured from the same devices following excitation with a CW laser at 532 nm.

**Fig. 2:**
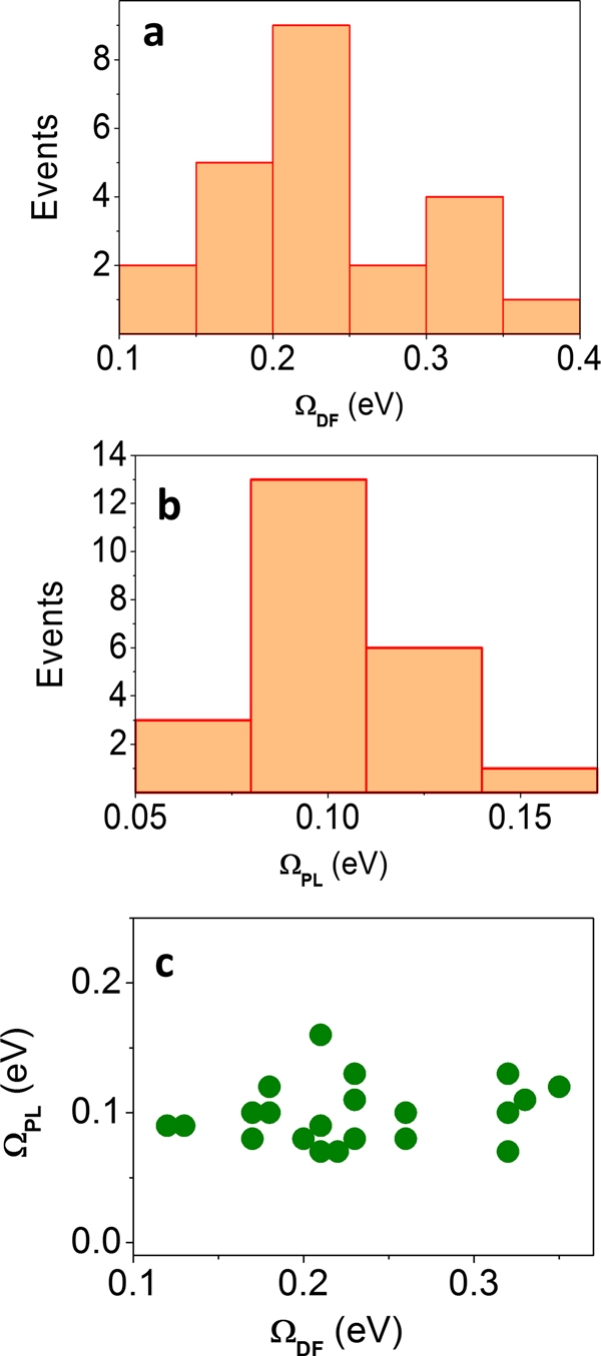
Peak splittings in scattering and PL. **a**, **b** Histograms of peak splitting values obtained from dark-field scattering spectra, Ω_DF_ (**a**) and from PL spectra, Ω_PL_ (**b**). **c** Correlation between splitting values in PL and in scattering.

Scattering and PL spectra were recorded from 23 bowtie cavities loaded with either one or a few QDs, and two examples are shown in Fig. 1b–e (see additional spectra in Supplementary Fig. 1). Scattering spectra show dips indicative of plasmon–exciton coupling^20^ (Fig. 1c, e, green lines). The splitting values obtained directly from the scattering spectra in Fig. 1 (i.e. the differences between the two peak positions) are 200 and 290 meV, respectively. Fits of the scattering spectra to a coupled-oscillator model^27,28^, presented in Supplementary Fig. 2, provide values for the coupling rate, *g*, of 52.6 ± 0.3 meV and 103.5 ± 1.1 meV, respectively. A histogram of the splitting values of all devices is shown in Fig. 2a, and a histogram of *g* values obtained from coupled-oscillator fits is shown in Supplementary Fig. 3. These values vary between 52.5 and 110 meV, with an average value of 71.7 meV.

It is instructive to ask where the coupling rates observed here places our PC-QD systems with respect to the strong-coupling regime. To that end, we compare our measured *g* values to two criteria often discussed in the literature^29^. The first criterion, *g* > (*γ*_p_ − *γ*_e_)/4 (where *g*, *γ*_p_ and *γ*_e_ are the coupling strength, plasmon linewidth and exciton linewidth, respectively), guarantees two real solutions in the coupled-oscillator model when the QD is resonantly tuned to the plasmon and can be interpreted as the definition of a lower bound for the strong-coupling regime. When this criterion is fulfilled the system has passed an exceptional point^30,31^ and is therefore guaranteed to possess two distinct eigenstates. The splitting in the spectrum above the exceptional point reflects the formation of two polaritonic states. Based on the values of *γ*_p_ and *γ*_e_ we measure on bare bowties and free QDs, respectively, the above criterion gives a threshold value of ~55 meV, and the vast majority of the values of *g* extracted from our spectra are larger.

While this criterion guarantees the existence of two eigenstates, it does not ensure that the measured spectra will clearly show these states as separate peaks. Therefore, a second and stricter criterion is often introduced. This criterion^29^, given by *g* > (*γ*_p_ + *γ*_e_)/4, is more heuristic and is connected with the establishment of Rabi oscillations in the time domain. In our case the latter criterion gives a value of ~120 meV, somewhat larger than the values we report here. However, as *g* increases from the limit given by the first criterion, splitting of the two modes in the spectrum grows continuously, and indeed splitting is systematically present in our experimental spectra. Therefore, we can safely state that the PC-QD systems measured here are found to be at the onset of the strong-coupling regime.

A peak splitting is also observed in PL spectra (Fig. 1c, e, red) recorded from the same cavities. PL spectra look significantly and consistently narrower than the corresponding scattering spectra, suggesting that different microscopic mechanisms account for splitting in the two cases. This difference is also manifested in the values of the splitting between peaks obtained from the PL spectra of Fig. 1c, e, which are only 100 and 110 meV, respectively. The histogram of the peak splitting values obtained from PL spectra is shown in Fig. 2b. Comparing this histogram to the one in Fig. 2a, we find that while in DF scattering spectra splitting values (Ω_DF_) are as high as 350 meV, the maximal splitting (Ω_PL_) observed in PL spectra is only 160 meV. A correlation plot of Ω_PL_ versus Ω_DF_ is shown in Fig. 2c. It is evident that the correlation is very weak, suggesting that Ω_PL_ does not depend on parameters like bowtie gap size, number of QDs and others to the same extent as Ω_DF_.

### HBT interferometry

**Fig. 3:**
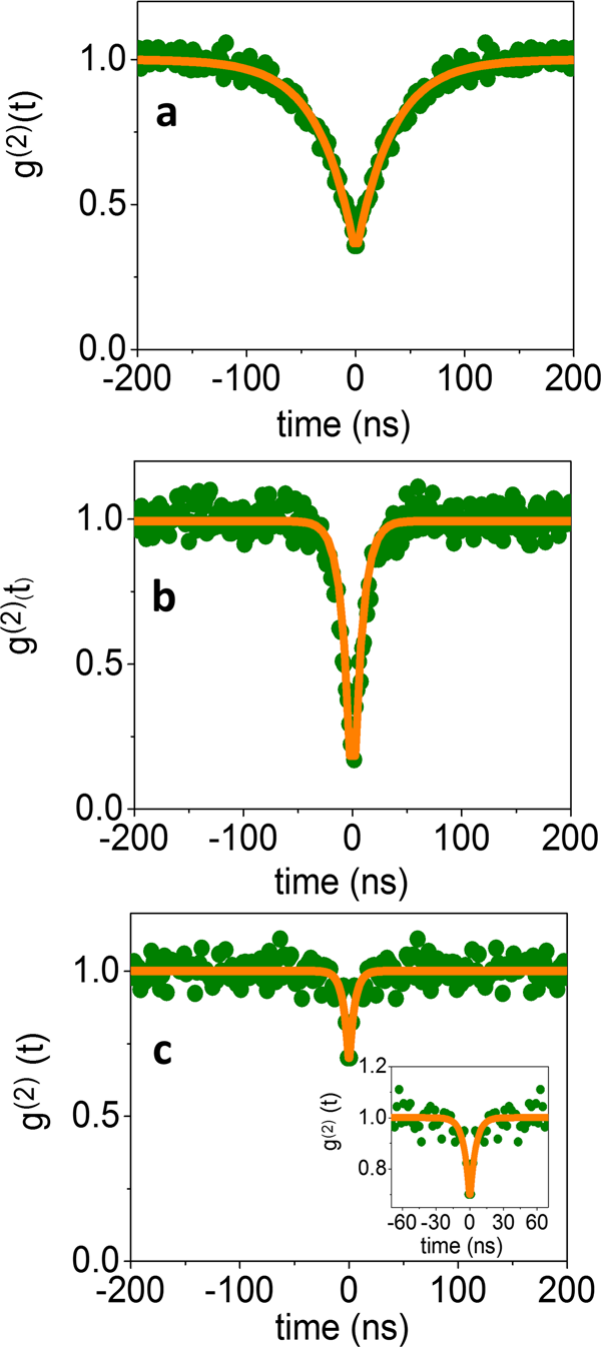
Second-order photon correlation function (*g*^(2)^(*t*)). **a** A bare QD on glass substrate. **b**, **c** QDs coupled to PCs. The dip at zero delay is a manifestation of a single QD in (**b**) and three QDs in (**c**). Green—experimental results, orange—fits to Eq. (1).

To shed light on the observation that Ω_PL_~ const < Ω_DF_ and to further understand the quantum properties of the light emitted by the coupled devices, we turned to HBT interferometry. We first measured the second-order photon correlation curves (*g*^(2)^(*t*)) of light emitted from individual QDs on a glass substrate. An example of such a correlation curve is shown in Fig. 3a. (Additional examples are presented in Supplementary Fig. 4.) The antibunching observed in the correlation curve at zero delay, with a value lower than 0.5, verifies that the measurement is indeed from a single QD. However, in some cases the number of QDs within the laser spot during the HBT measurement was larger than one. In order to obtain both the lifetime of the emitting exciton and the number of QDs, we therefore fitted the measured correlation curves with Eq. (1),

1 $$g^{(2)}\left( t \right) = A + B\,(1 - e^{ - |\frac{t}{\tau }|}).$$

In this equation, A and B are constants and τ is the lifetime of the emitting exciton (i.e. the total decay time). The value of the second-order photon correlation curves at zero time delay, *g*^(2)^(0), scales as 1 − 1/*N*, where N is the number of QDs^32^. However, background photons reduce slightly the zero time dip, which is given by the constant A obtained from the fit. The maximal possible *N* based on a particular *g*^(2)^(0) measurement is the largest integer smaller than 1/(1−*A*). We obtained 22 correlation curves of individual QDs on glass, and used these in order to plot the distribution of exciton lifetimes, which arises due to their non-uniform size distribution (Supplementary Fig. 4). The average exciton lifetime is 24 ns, and the distribution is asymmetric with a standard deviation of 5.3 ns.

We then measured the second-order photon correlation curves from QDs within PCs. Two examples are shown in Fig. 3b, c. As in the case of QDs on glass, these correlation curves show clear evidence of antibunching, pointing to the non-classical nature of the emitted light. Fitting the correlation curves to Eq. (1), we found that the probed devices contained one QD (Fig. 3b) and three QDs (Fig. 3c). The fits also provided the polariton lifetimes for the two devices: 5.6 ± 0.3 ns and 3.5 ± 0.2 ns, respectively. Overall, *g*^(2)^(*t*) functions were measured from 16 of the devices whose scattering and PL spectra showed a clear indication of peak splitting. More examples of *g*^(2)^(*t*) are provided in Supplementary Fig. 5, whose panel h shows the distribution of lifetimes obtained from fits to the correlation curves, ranging from 3 to 12 ns. Surprisingly, there seems to be only a minor shortening of the lifetimes (by a factor of ~5) compared to QDs on glass. To verify this result, we also performed direct time-resolved PL measurements of several devices, the results of which are shown in Supplementary Fig. 6. The average lifetimes extracted from these measurements are also shortened by a factor of ~5 only compared to the lifetimes of bare QDs (see Supplementary Table 1). This finding is highly unexpected, as the mixing of the QD exciton with the plasmon in the cavity should have opened a fast relaxation channel with a lifetime closer to that of the plasmon^33^. A recent study of an ensemble of QDs deposited on a plasmonic hole array also reported only a modest shortening of the excited-state lifetime^34^. Interestingly, Ebbesen and coworkers found a similar deviation from the expected shortening of the PL lifetime in a different system consisting of molecules coupled to a microcavity^9^.

### Extended Jaynes–Cummings model: beyond two levels

Three surprising observations emerge from the experiments reported above. First, PL spectral peaks of QDs coupled to the PCs are narrower than those in scattering spectra. Second, the splitting between peaks observed in PL spectra is not correlated with the splitting in scattering spectra. Finally, the PL lifetime seems to be only mildly shortened compared to that of QDs on glass. All three observations deviate from expectations for strongly or close-to-strongly coupled QD-plasmonic devices. Indeed, the formation of polaritonic states due to coupling, as described within a standard Jaynes–Cummings Hamiltonian of a two-level system coupled to an optical cavity, would lead to (i) broad scattering and PL spectral peaks, (ii) correlation between the values of splitting seen in the two spectra, and (iii) PL lifetimes on the femtosecond time scale, close to the ultrafast decay times of the PCs. Therefore, the spectral features and the decay of the PL found here indicate a picture that is significantly more complex than that described with a simple coupled two-level model. Indeed, the presence of long-lived dark excitonic states of different origins in QDs has been reported in the literature^35–37^. We thus consider the role of a dark state of the QDs as a key contributor to the observed excited-state dynamics of the coupled system.

**Fig. 4:**
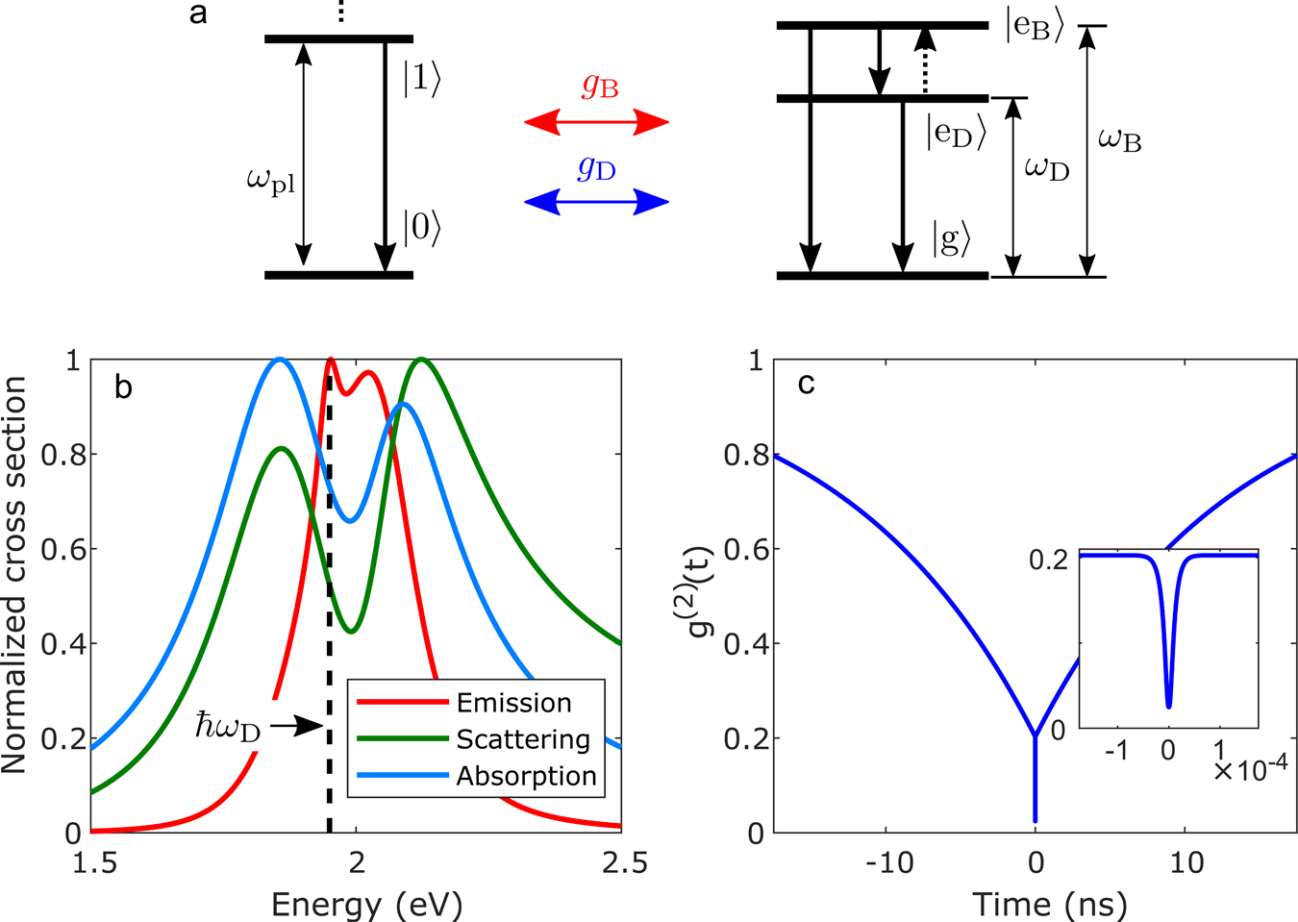
Quantum simulations of the plasmon-QD coupling dynamics. **a** Schematic level diagram describing the theoretical model. The plasmonic cavity is depicted on the left with an excited state of energy $\omega _{pl}.$ The QD (right) is described as a three-level electronic system containing a ground state $|{\mathrm{g}}\rangle$, a bright excitonic level, $|{\mathrm{e}}_{\mathrm{B}}\rangle$, and a dark excitonic level, $|{\mathrm{e}}_{\mathrm{D}}\rangle$. The bright (dark) excitonic transition occurs at energy $\omega _{\mathrm{B}}$($\omega _{\mathrm{D}}$). For the PL spectrum. we assume that both the bright and the dark excitons are pumped incoherently. Plasmon–exciton coupling is described within the Jaynes–Cummings model with rates $g_{\mathrm{B}}$ and $g_{\mathrm{D}}$ (for coupling of the bright and dark exciton, respectively). A detailed description of the model terms is provided in the Methods section, and the parameters used are given in Table 1. **b** Emission (red), scattering (green), and absorption (blue) spectra calculated theoretically for parameters shown in Table 1. The dashed line marks the energy of the dark exciton, $\hbar \omega _{\mathrm{D}}$. **c** A simulated *g*^(2)^(*t*) features a two-component decay. Inset shows a zoom of the fast (fs) decay of the system excitations that is not resolved on the ns time scale.

To simulate such dynamics and examine their potential effect on the experimental observations, we adopt a cavity-quantum electrodynamics (c-QED) theoretical framework. We extend the Jaynes–Cummings Hamiltonian beyond the standard two-level description and include a weakly coupled dark state. We also add Lindblad terms to the Hamiltonian in order to describe incoherent pumping (for the PL spectra) and all the corresponding relaxation channels. The quantum emitter is therefore modelled as an electronic system composed of three levels: a ground state, a level with a large decay rate into the plasmon, representing the bright excitonic state, and another level, positioned slightly lower in energy and possessing a much smaller (yet non-zero) decay rate, representing the dark state. A scheme of the plasmon and quantum emitter energy levels used in this model is shown in Fig. 4a, and the relevant parameters (selected to properly describe the physical properties of the system) are given in Table 1. From dynamic simulations based on this model, we calculate scattering and PL spectra, as well as second-order photon correlation functions, which are shown in Fig. 4b and c for a representative case. The relative lack of dependence of these spectra and correlations on the value selected for the intrinsic decay rate of the dark exciton is demonstrated in Supplementary Fig. 7.

**Table 1:** Set of parameters used to reproduce spectra and $g^{(2)}\left( t \right)$ functions, as shown in Fig. 4.

$\hbar {\upkappa}$	400 meV	Intrinsic plasmon decay rate
$\hbar {\upgamma}_{{\mathrm{gB}}}$	0.1 $\upmu {\mathrm{eV}}$	Intrinsic decay rate of the bright exciton
$\hbar {\upgamma}_{{\mathrm{gD}}}$	$0.005\,\upmu {\mathrm{eV}}$	Intrinsic decay rate of the dark exciton
$\hbar {\upgamma}_{{\mathrm{DB}}}^0$	0.2 $\upmu {\mathrm{eV}}$	Rate of energy transfer between the dark and the bright exciton
$\hbar {\upgamma}_{{\mathrm{BB}}}$	130 meV	Pure dephasing of the bright exciton
$\hbar {\upgamma}_{{\mathrm{DD}}}$	50 meV	Pure dephasing of the dark exciton (broadening of the dark-exciton line)
$\hbar {\upgamma}_{{\mathrm{Bg}}}$	1 n${\mathrm{eV}}$	Incoherent pumping of the bright exciton
$\hbar {\upgamma}_{{\mathrm{Dg}}}$	5 n${\mathrm{eV}}$	Incoherent pumping of the dark exciton
$\hbar {\mathrm{g}}_{\mathrm{B}}$	100 meV	Coupling between plasmon and the bright exciton
$\hbar {\mathrm{g}}_{\mathrm{D}}$	35 $\upmu {\mathrm{eV}}$	Coupling between plasmon and the dark exciton
$\hbar {\upomega}_{{\mathrm{pl}}}$	1.93 ${\mathrm{eV}}$	Plasmon energy
$\hbar {\upomega}_{\mathrm{D}}$	1.95 ${\mathrm{eV}}$	Emission energy of the dark exciton
$\hbar {\upomega}_{\mathrm{B}}$	2.00 ${\mathrm{eV}}$	Emission energy of the bright exciton

The choice of parameters has been guided by the experimental results.

Importantly, the simulated second-order photon correlation curves (Fig. 4c and Supplementary Fig. 8) involve two distinct decay components, a very fast one on the femtosecond time scale, and a much slower one, on the nanosecond time scale. The fast decay component is related to the dynamics of the bright exciton, modified by the involvement of the plasmonic decay channels. However, the time scale of this component is too short to be observed in our experiments. Hence, only the slow decay component of the correlation curve is registered experimentally. This component can be attributed to the decay of the dark state into two possible channels. The first decay channel is due to the population transfer to the bright excitonic mode of the QD, from which fast emission brings the system back to the ground state. The second decay channel involves the enhanced emission due to weak coupling of the dark state to the plasmonic mode. The effect of the coupling of the bright and dark excitons to the plasmon on the resolution of the fast component of *g*^(2)^ is illustrated in Supplementary Fig. 9.

**Fig. 5:**
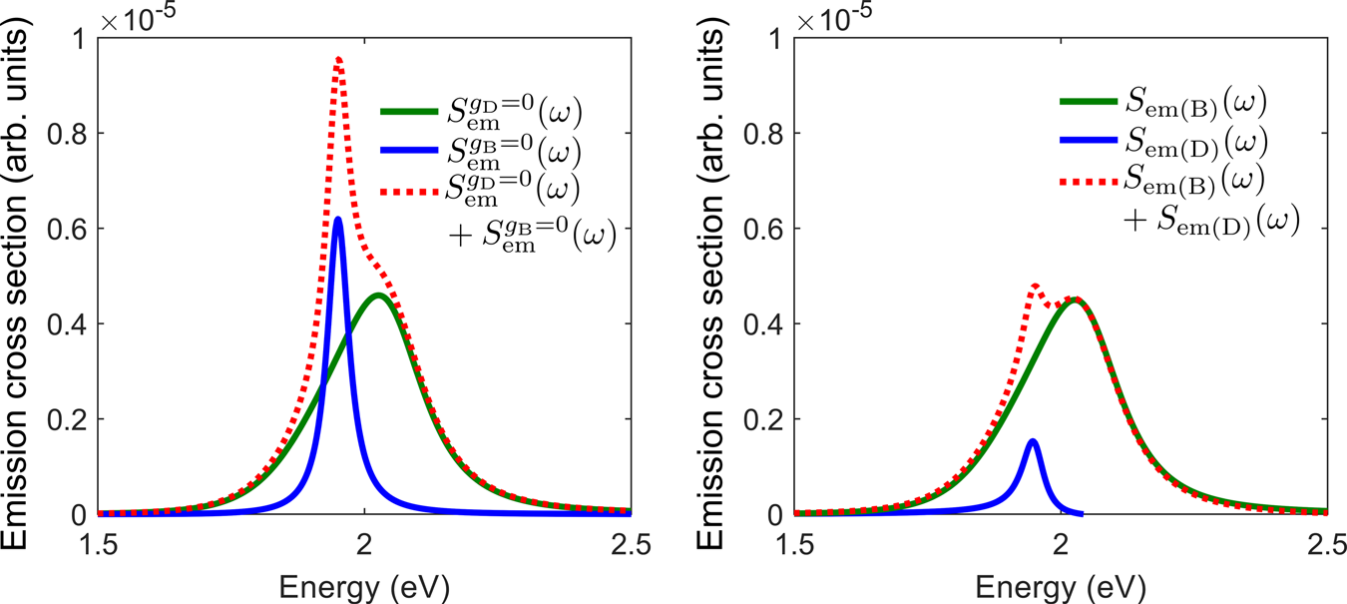
Quantum simulations of the plasmon-QD emission spectrum. **a** Numerically calculated emission spectra of the system for $g_{\mathrm{D}} = 0$, $S_{{\mathrm{em}}}^{g_{\mathrm{D}} = 0}\left( \omega \right)$ (green line), for $g_{\mathrm{B}} = 0$, $S_{{\mathrm{em}}}^{g_{\mathrm{B}} = 0}\left( \omega \right)$ (blue line), and sum of the two, $S_{{\mathrm{em}}}^{g_{\mathrm{D}} = 0}\left( \omega \right) + S_{{\mathrm{em}}}^{g_{\mathrm{B}} = 0}\left( \omega \right)$ (red dotted line). **b** Analytically calculated contributions to the emission spectrum due to the bright exciton, $S_{{\mathrm{em}}\left( {\mathrm{B}} \right)}\left( \omega \right)$ (green line), dark exciton, $S_{{\mathrm{em}}\left( {\mathrm{D}} \right)}\left( \omega \right)$ (blue line), and the total analytical spectrum containing both contributions, $S_{{\mathrm{em}}}\left( \omega \right) = S_{{\mathrm{em}}\left( {\mathrm{B}} \right)}\left( \omega \right) + S_{{\mathrm{em}}\left( {\mathrm{D}} \right)}\left( \omega \right)$ (red dotted line).

The calculated PL spectra are significantly narrower than the scattering spectra (Fig. 4b), and show a reduced splitting between their emission peaks, in qualitative agreement with the experimental observations. To understand the origin of the two peaks in the PL spectra and to clarify how the bright exciton influences the direct emission from the dark exciton, we perform a series of calculations in which some of the coupling channels are cancelled. We first numerically calculate the photoluminescence spectra for two tailored scenarios in which either the dark exciton or the bright exciton is decoupled from the plasmon. Importantly, while in each scenario only one of the excitons couples to the plasmon, both excitons are still allowed to incoherently exchange populations via incoherent decay and pumping processes. We show in Fig. 5a the PL spectrum calculated with a decoupled dark exciton, $S_{{\mathrm{em}}}^{g_{\mathrm{D}} = 0}\left( \omega \right)$ (green line) and with a decoupled bright exciton, $S_{{\mathrm{em}}}^{g_{\mathrm{B}} = 0}\left( \omega \right)$ (blue line). $S_{{\mathrm{em}}}^{g_{\mathrm{B}} = 0}\left( \omega \right)$ features a single peak due to emission from the dark exciton. Remarkably, $S_{{\mathrm{em}}}^{g_{\mathrm{D}} = 0}\left( \omega \right)$ also shows a single (asymmetric) peak, a signature of the bright-exciton emission at the onset of the strong-coupling regime^23^. This peak is broader than the original exciton peak due to the interaction with the cavity plasmon. As the system is at the onset of strong coupling, the spectral feature appears as an asymmetric peak due to the influence of the not-fully developed upper polariton. If we directly sum the two contributions ($S_{{\mathrm{em}}}^{g_{\mathrm{D}} = 0}\left( \omega \right) + S_{{\mathrm{em}}}^{g_{\mathrm{B}} = 0}\left( \omega \right)$, red dotted line), we can observe that the dark-exciton peak becomes a prominent sharp feature on top of the broader bright-exciton peak. This result strongly differs from the calculation based on the full model, where all the couplings between QD and PC states are considered (shown in Fig. 4b, red line). This indicates that the simple sum of uncoupled emissions, $S_{{\mathrm{em}}}^{g_{\mathrm{D}} = 0}\left( \omega \right) + S_{{\mathrm{em}}}^{g_{\mathrm{B}} = 0}\left( \omega \right)$, cannot fully capture the underlying physics of the emission.

We next show in Fig. 5b an analytical decomposition of the total PL spectrum, into the contribution from the bright-exciton, $S_{{\mathrm{em}}\left( {\mathrm{B}} \right)}\left( \omega \right)$ (green line), and that from the dark exciton, $S_{{\mathrm{em}}\left( {\mathrm{D}} \right)}\left( \omega \right)$ (blue line), while maintaining the couplings *g*_B_ and *g*_D_ (see details of the model and explicit expressions of this decomposition in Supplementary Note). The total spectrum given by the sum of both contributions, $S_{{\mathrm{em}}}\left( \omega \right) = S_{{\mathrm{em}}\left( {\mathrm{B}} \right)}\left( \omega \right) + S_{{\mathrm{em}}\left( {\mathrm{D}} \right)}\left( \omega \right)$ (red dotted line), agrees very well with the numerical result shown in Fig. 4b (see a direct comparison in Supplementary Fig. 10).

The contribution of the dark exciton, $S_{{\mathrm{em}}\left( {\mathrm{D}} \right)}\left( \omega \right)$, is strongly affected by the bright-exciton coupling. When this coupling is switched on [blue line in Fig. 5b], the emission of the dark exciton is dramatically reduced compared to the $g_{\mathrm{B}} = 0$ case [blue line in Fig. 5a]. This is due to the formation of hybrid states involving the bright exciton and the plasmon, and to the resulting modulation of the photonic density of states (PDOS), which leads to a lower probability of light emission from the dark exciton and thus reduces the intensity of the dark-exciton contribution. On the other hand, we observe that the emission of the bright exciton is not affected by the coupling of the dark exciton to the cavity [compare green lines for $S_{{\mathrm{em}}}^{g_{\mathrm{D}} = 0}\left( \omega \right)$ in Fig. 5a and $S_{{\mathrm{em}}\left( {\mathrm{B}} \right)}\left( \omega \right)$ in Fig. 5b], since the dark exciton only weakly perturbs the plasmonic response. Nevertheless, the presence of the dark exciton still indirectly affects the strength of the bright plasmon, due to incoherent and pumping processes intrinsic to the emitter (included in both models).

We have thus shown that the bright exciton and the dark exciton contribute to the PL spectra in two distinct ways. The complex dynamics of the bright exciton, non-perturbatively coupled to the plasmonic cavity, leads to the formation of an asymmetric emission peak whose line shape deviates from a simple Lorentzian profile. The amplitude of this peak depends on the incoherent pumping of the bright exciton by the external illumination, either directly or via the dark exciton. On the other hand, the dark exciton couples to the plasmonic cavity only weakly, and contributes to the light emission indirectly, via incoherent pumping of the bright exciton, and directly via weak coupling with the plasmonic cavity. Due to the weak perturbative character of the dark-exciton coupling, its direct contribution to the light emission, properly modified by the bright state, can be superimposed on top of the photoluminescence spectrum of the bright exciton (also modified by the dark-state pumping). This theoretical model thus shows that the emission probability of the dark exciton via the plasmon is strongly modified due to the modulation of the PDOS by the coupling of the plasmon and the bright exciton. Our results thus indicate that it is essential to consider all coupling and decay channels for a full description of the dynamics and emission properties of the system.

## Discussion

We reported here a vast set of measurements of QDs embedded within PCs, which allowed us to expose unique excited-state dynamics involving complex interaction between bright and dark states. Coupling values of 50–110 meV were deduced from light-scattering spectra by fitting to a coupled-oscillator model, indicating that our devices are close to the strong-coupling limit. In addition to scattering, we also obtained the PL spectrum of each device, and for most devices we also recorded the second-order photon correlation curves. The observation of antibunching in correlation curves demonstrated unequivocally the non-classical nature of the light emitted by these devices, which originated from either one or just a few (countable) QDs. Our PL measurements revealed several deviations from expectations based on the familiar picture of a simple two-level quantum emitter coupled to a cavity resonance. An extended Jaynes–Cummings model that explicitly took into account the presence of a dark state in the QD within a c-QED framework nicely accounts for all the intriguing features in the experiments. This quantum model allowed us to show how the interplay of the weak effect of the PC on the dark exciton and the much stronger effect on the bright exciton leads to the complex dynamics exposed in our experiments.

Theoretical and experimental studies of QDs have revealed different types of dark states. Exchange interactions lead to the splitting of the band-edge exciton with the appearance of a dark state as the lowest energy level and a bright state above it^35^. Experimental work provided direct evidence for this splitting and showed that the dark and bright states are separated by less than 1 meV^38^. This energy difference is too small to account for our observations. On the other hand, the occurrence of trapped surface states whose transitions are significantly red-shifted compared to the bright exciton^36,37^ can account for the hierarchy of energies used in our model. A location of a few tens of meV to the red side of the PL peak for the surface trap state has already been discussed in the literature, e.g. by Morello et al.^39^. Bradley and coworkers also modelled their experimental data with a dark trap state, though they assign a smaller value of 4–7 meV to the shift of the trap state from the main luminescent state^40^. This value is likely dependent on the particular type of QDs studied, and might in reality be distributed over a certain range. We are not aware of any additional excited states of QDs whose involvement might explain our results. A high-energy shoulder on the PL spectrum of a coupled QD was reported in ref. ^23^, and was proposed to be due to a charged exciton or multiexcitonic states. While this high-energy shoulder is likely due to a different origin than the low-energy peak in our spectra, one cannot completely discard the possibility that a similar mechanism contributes to the current results. Nevertheless, our theoretical simulations support the assertion that the low-energy narrow emission line is related to a dark state; the interaction of this dark state with the plasmonic cavity dramatically enhances its emission. This enhancement is still influenced by the plasmon coupling to the bright state, which modulates the final exact contribution of the dark state to the total photoluminescence.

Our findings, based on joint experimental and theoretical observations, demonstrate unexpectedly rich excited-state dynamics induced by coupling of a small number of quantum emitters to a PC. The ability to access and eventually control this complex dynamics of excited states in light emitters can pave the way for manipulation of electronic excitations at room temperature in strongly coupled devices. This is a necessary first step for future applications, such as the construction of quantum devices operating under ambient conditions and the modulation of chemical reactivity at the single-molecule level.

## Methods

### Fabrication of silver bowties

SiN grids (TEM windows) were cleaned with plasma (O_2_ ~ 3.5 sccm and Ar ~ 1.5 sccm) at 150 W. The cleaned grids were spin-coated with polymethylmethacrylate (PMMA) at 4000 r.p.m. for 45 s to get a 60 nm thick layer of the polyemer, followed by baking at 180 °C for 90 s. The PMMA coated grids were then transferred to a Raith E_line Plus electron-beam lithography chamber for electron-beam exposure of PMMA in a series of pre-defined bowtie shapes, using an accelerating voltage of 30 kV and a current of 30 pA. The overall design of each fabricated grid involved matrices of bowties that were separated by 10 µm from each other to avoid any potential interaction between them. Each bowtie was composed of two 80 nm equilateral triangles, so that its plasmon resonance overlapped with the QD emission frequency (see Supplementary Fig. 11 for the scattering and PL spectra of an empty bowtie and a QD). The exposed PMMA was developed in a solution containing methyl isobutyl ketone and isopropanol in 1:3 ratio for 30 s, followed by dipping in isopropanol (stopper) for 30 s and drying in a N_2_ gas flow. Subsequently, 3 nm chromium was deposited as an adhesion layer, which was then followed by evaporation of a 20 nm silver layer within an electron-beam evaporator (Odem Scientific Applications). Following metal deposition, a liftoff process was carried out using a REMOVER PG solvent stripper (MicroChem) to obtain a set of silver bowties on the SiN grid.

### Incorporation of QDs into the gap regions of bowties

The resist ZEP (a 1:1 copolymer of α-chloromethacrylate and α-methylstyrene) was spin-coated on the bowtie sample at 3000 r.p.m. for 45 s, and the sample was then baked for 180 s at 180 °C. By using alignment marks, the electron beam was positioned at the bowtie gaps with an overlay accuracy of a few nm to generate holes in the resist. The exposed regions were developed in amyl acetate and isopropanol. In order to drive QDs into the holes, we followed a method developed by Alivisatos and colleagues^41^. The sample was placed vertically in an aqueous solution of QDs, and the solvent was allowed to evaporate slowly, exerting a capillary force along the receding line of contact, which drove the QDs into the holes. The number of QDs in the gap region could be partly controlled to be one, two or many by tuning the concentration of QD solution and diameter of the holes. A schematic of the bowtie fabrication and QD trapping process is shown in Fig. 1a.

### Dark-field and PL microspectrometry

Scattering spectra were measured using a home-built setup based on an inverted microscope and equipped with a 75 W Xenon lamp (Olympus), a dark-field condenser, a ×100 oil immersion objective of a tunable numerical aperture (from 0.9 to 1.3), a 150 mm spectrograph (SpectraPro-150, Acton) and an air-cooled CCD camera (Newton, Andor Technologies). A NA of 0.9 was typically used in these experiments. PL measurements were performed on the same setup using a NA of 1.3. The excitation source was a 532 nm laser, whose polarization was selected to be parallel to the long axes of the bowties. All the spectra were smoothed with a Savitzky-Golay filter.

### Time-resolved PL measurements

HBT interferometry and PL decay measurements were carried out using a Micro Time 200 (PicoQuant) single-molecule spectrometer. A 485 nm CW laser was used to excite the QDs through a ×60 water-immersion objective. For second-order correlation measurements, emitted light from the sample was collected with the same objective and passed through a 50/50 beam splitter before being focused on two single-photon avalanche photodiodes (Excelitas). A band-pass filter was inserted in front of each detector to reduce the unwanted background signal. A single detector was used for time-resolved single-photon counting measurements with the same system. In both types of measurement, we used a HydraHarp 400 time-interval analyzer (PicoQuant) for signal registration.

### Scanning transmission electron microscopy (STEM)

Plasmonic bowties and QDs were imaged using a Zeiss Gemini SEM microscope in a STEM mode, with an electron-beam energy of 30 keV, a 20 μm aperture and a 5 mm working distance.

### Theoretical model

We assume that a quantum dot is composed of a ground state $|{\mathrm{g}}\rangle$ of energy $E_{\mathrm{g}}$= 0 eV, a bright excitonic level, $|{\mathrm{e}}_{\mathrm{B}}\rangle$, of energy $E_{\mathrm{B}} = E_{\mathrm{g}} + \hbar \omega _{\mathrm{B}}$, and a dark level, $|{\mathrm{e}}_{\mathrm{D}}\rangle$, of energy $E_{\mathrm{D}} = E_{\mathrm{g}} + \hbar \omega _{\mathrm{D}}$. The Hamiltonian describing the QD,$H_{{\mathrm{QD}}}$, can be expressed as:

2 $${\mathrm{H}}_{{\mathrm{QD}}} = \hbar {\upomega}_{\mathrm{B}} {| {{\mathrm{e}}_{\mathrm{B}}}} \rangle \langle {{\mathrm{e}}_{\mathrm{B}}} | + \hbar {\upomega}_{\mathrm{D}}| {{\mathrm{e}}_{\mathrm{D}}}\rangle\langle{{\mathrm{e}}_{\mathrm{D}}} |$$

The QD is coupled to a single mode of a plasmonic cavity of energy $\hbar \omega _{{\mathrm{pl}}}$, described via the Hamiltonian $H_{{\mathrm{pl}}}$:

3 $$H_{pl} = \hbar \omega _{{\mathrm{pl}}}a^\dagger a$$

where $a$ $\left( {a^\dagger } \right)$ is a bosonic annihilation (creation) operator. The plasmon–exciton coupling is included via the Jaynes–Cummings coupling terms:

4 $$H_{{\mathrm{pl}} - {\mathrm{QD}}} = \hbar g_{\mathrm{B}}(a^\dagger \left| {\mathrm{g}}\rangle \right.\langle {\mathrm{e}}_{\mathrm{B}}| + a\left| {{\mathrm{e}}_{\mathrm{B}}}\rangle \right.\langle {\mathrm{g}}|) + \hbar g_D(a^\dagger \left| {\mathrm{g}}\rangle \right.\langle {\mathrm{e}}_{\mathrm{D}}| + a\left| {{\mathrm{e}}_{\mathrm{D}}}\rangle \right.\langle {\mathrm{g}}|),$$

where $g_{\mathrm{B}}$ ($g_{\mathrm{D}}$) is the Jaynes–Cummings constant coupling the bright (dark) level to the plasmon.

The total Hamiltonian, $H$, of the system thus becomes

5 $$H = H_{{\mathrm{QD}}} + H_{{\mathrm{pl}}} + H_{{\mathrm{pl}} - {\mathrm{QD}}}.$$

To obtain the observables of the system, we solve the Liouville–von Neumann equation for the system’s density matrix, $\rho$:

6 $$\frac{d}{{dt}}\rho = - \frac{i}{\hbar }\left[ {H,\;\rho } \right] + \mathop {\sum }\limits_{\mathrm{i}} {\upgamma}_{{\mathrm{O}}_{\mathrm{i}}}{\cal{L}}_{O_i}\left[ \rho \right]$$

which includes incoherent Lindblad operators, added to account for losses and pure dephasing. These operators take the following form:

7 $$\gamma _{{\mathrm{O}}_i}{\cal{L}}_{{\mathrm{O}}_i}\left[ \rho \right] = \frac{{\gamma _{{\mathrm{O}}_i}}}{2}(2{\mathrm{O}}_i\rho {\mathrm{O}}_i^\dagger - \{ {\mathrm{O}}_i^\dagger {\mathrm{O}}_i,\rho \} ),$$

where $O_i$ is a generic system operator to be specified, and $\dagger$ stands for Hermitean conjugate. In particular, we add the following Lindblad terms:

8 $${\kappa}{\cal{L}}_a\left[ {\rho} \right]\, \left( {{\mathrm{Plasmonic}}\,{\mathrm{decay}}} \right),$$

9 $${\gamma}_{{\mathrm{gB}}}{\cal{L}}_{\left| {\mathrm{g}}\rangle \right.\langle {\mathrm{e}}_{\mathrm{B}}|}\left[ {\rho} \right]\, \left( {{\mathrm{Decay}}\,{\mathrm{of}}\,{\mathrm{the}}\,{\mathrm{bright}}\,{\mathrm{excitonic}}\,{\mathrm{level}}} \right),$$

10 $${\gamma}_{{\mathrm{gD}}}{\cal{L}}_{\left| {\mathrm{g}}\rangle \right.\langle {\mathrm{e}}_{\mathrm{D}}|}\left[ {\rho} \right]\, \left( {{\mathrm{Decay}}\,{\mathrm{of}}\,{\mathrm{the}}\,{\mathrm{dark}}\,{\mathrm{excitonic}}\,{\mathrm{level}}} \right),$$

11 $${\gamma}_{{\mathrm{BD}}}{\cal{L}}_{\left| {{\mathrm{e}}_{\mathrm{B}}}\rangle \right.\langle {\mathrm{e}}_{\mathrm{D}}|}\left[ {\rho} \right]\, \left( {{\mathrm{Population}}\,{\mathrm{transfer}}\,{\mathrm{from}}\,{\mathrm{the}}\,{\mathrm{dark}}\,{\mathrm{level}}\,{\mathrm{into}}\,{\mathrm{the}}\,{\mathrm{bright}}\,{\mathrm{level}}} \right),$$

12 $${\gamma}_{{\mathrm{DB}}}{\cal{L}}_{\left| {{\mathrm{e}}_{\mathrm{D}}}\rangle \right.\langle {\mathrm{e}}_{\mathrm{B}}|}\left[ {\rho} \right]\, \left( {{\mathrm{Decay}}\,{\mathrm{of}}\,{\mathrm{the}}\,{\mathrm{bright}}\,{\mathrm{level}}\,{\mathrm{into}}\,{\mathrm{the}}\,{\mathrm{dark}}\,{\mathrm{level}}} \right),$$

13 $${\gamma}_{{\mathrm{DD}}}{\cal{L}}_{\left| {{\mathrm{e}}_{\mathrm{D}}}\rangle \right.\langle {\mathrm{e}}_{\mathrm{D}}|}\left[ {\rho} \right]\, \left( {{\mathrm{Pure}}\,{\mathrm{dephasing}}\,{\mathrm{of}}\,{\mathrm{the}}\,{\mathrm{dark}}\,{\mathrm{level}}} \right),$$

14 $${\gamma}_{{\mathrm{BB}}}{\cal{L}}_{\left| {{\mathrm{e}}_{\mathrm{B}}}\rangle \right.\langle {\mathrm{e}}_{\mathrm{B}}|}\left[ {\rho} \right]\, \left( {{\mathrm{Pure}}\,{\mathrm{dephasing}}\,{\mathrm{of}}\,{\mathrm{the}}\,{\mathrm{bright}}\,{\mathrm{level}}} \right).$$

Furthermore, we assume that the process of population transfer from the dark state to the bright state (and vice versa) is thermally activated and hence we get:

15 $${\gamma}_{{\mathrm{DB}}} = \left[ {1 + N_{{\mathrm{th}}}\left( {\hbar \omega _{\mathrm{B}} - \hbar \omega _{\mathrm{D}};T} \right)} \right]\gamma _{{\mathrm{DB}}}^0$$

16 $${\gamma}_{{\mathrm{BD}}} = N_{{\mathrm{th}}}\left( {\hbar \omega _{\mathrm{B}} - \hbar \omega _{\mathrm{D}};T} \right)\gamma _{{\mathrm{DB}}}^0$$

where $N_{{\mathrm{th}}}\left( {E;T} \right)$ is the Bose–Einstein distribution at temperature $T$ (we assume $T = 300$ K) and energy $E$ and $\gamma _{{\mathrm{DB}}}^0$ is the spontaneous decay rate of the bright state, $\left| {{\mathrm{e}}_{\mathrm{B}}}\rangle \right.$, into the dark state, $\left| {{\mathrm{e}}_{\mathrm{D}}}\rangle \right.$. This rate, $\gamma _{{\mathrm{DB}}}^0$, would be likely due to phonon-mediated processes that can lead to inter-exciton relaxation faster than the excitonic spontaneous emission^42^. The exact value of this rate would depend on the microscopic details of the electron–phonon interaction in a specific quantum dot, for which a valid estimate is very challenging to obtain^43–45^.

In the PL calculations, we assume that the bright state, $\left| {{\mathrm{e}}_{\mathrm{B}}}\rangle \right.$, as well as the dark state, $\left| {{\mathrm{e}}_{\mathrm{D}}}\rangle \right.$, are incoherently pumped via terms ${\upgamma}_{{\mathrm{Bg}}}{\cal{L}}_{\left| {{\mathrm{e}}_{\mathrm{B}}}\rangle \right.\langle {\mathrm{g}}|}\left[ \rho \right]$ and ${\upgamma}_{{\mathrm{Dg}}}{\cal{L}}_{\left| {{\mathrm{e}}_{\mathrm{D}}}\rangle \right.\langle {\mathrm{g}}|}\left[ \rho \right]$, respectively. This accounts for pumping of the QD states via another higher-energy bright state that is directly excited by an incident monochromatic laser. In the calculation of absorption and scattering, linear response theory is applied.

### Calculation of spectra

We calculate the absorption ${\mathrm{S}}_{{\mathrm{abs}}}\left( {\upomega} \right)$, scattering ${\mathrm{S}}_{{\mathrm{sca}}}\left( {\upomega} \right)$ and emission $S_{{\mathrm{em}}}\left( \omega \right)$ spectra using the following formulas, valid close to the plasmonic resonance:

17 $$S_{{\mathrm{abs}}}\left( \omega \right) \propto \omega {\mathrm{Re}}\left\{ {\mathop {\smallint }\limits_0^\infty \langle a(t)a^\dagger (0)\rangle e^{i\omega t}dt} \right\},$$

18 $$S_{{\mathrm{sca}}}\left( \omega \right) \propto \omega ^4\left| {\mathop {\smallint }\limits_0^\infty \langle a(t)a^\dagger (0)\rangle e^{i\omega t}dt} \right|^2,$$

19 $$S_{{\mathrm{em}}}\left( \omega \right) \propto \omega ^4{\mathrm{Re}}\left\{ {\mathop {\smallint }\limits_0^\infty \langle a^\dagger (0)a(t)\rangle e^{i\omega t}dt} \right\},$$

Here we assume that the system absorbs, scatters and emits light predominantly via the plasmonic cavity and neglect any direct absorption, scattering or emission of the quantum dot.

We evaluate the two-time correlation functions $\langle a(0)a^\dagger (t)\rangle$ and $\langle a(t)a^\dagger (0)\rangle$ using the quantum regression theorem (QRT) as described elsewhere^46^.

The second-order photon correlation function $g^{(2)}(t)$ is evaluated in the framework of cavity-quantum electrodynamics from the QRT as:

20 $$g^{(2)}\left( t \right) = \frac{{\langle a^\dagger \left( 0 \right)a^\dagger \left( t \right)a\left( t \right)a\left( 0 \right) \rangle}}{{\langle a^\dagger a\rangle}^2 }.$$

### Analytical model for the excitonic photoluminescence spectrum

We calculate the effective dynamics of the dark and the bright excitons with the use of an analytical model that we outline next. The explicit details can be found in the Supplementary Note. For the dark exciton, we eliminate the plasmonic cavity interacting with the bright exciton using the adiabatic approximation and obtain effective decay rates due to the cavity-induced Purcell effect $\gamma _{{\mathrm{Pur}}}^{\mathrm{D}}$. In this derivation, we use the separation of time scales present in the system due to the condition $g_{\mathrm{B}} \gg g_{\mathrm{D}}$ and show that the interaction of the dark exciton with the cavity is strongly influenced by the presence of the bright exciton, which significantly modifies the cavity response. The coupling of the bright exciton with the cavity, on the other hand, can be affected by the presence of the dark exciton via the incoherent pumping from the dark exciton, $\gamma _{{\mathrm{BD}}}$, and to a smaller degree by the decay from the bright exciton into the dark one, $\gamma _{{\mathrm{DB}}}$. These incoherent processes barely influence the shape of the bright-exciton photoluminescence spectrum, but they can significantly contribute to its emission intensity. To derive the effective decay rate of the dark exciton and the steady-state populations of the dark exciton, $\sigma _{{\mathrm{DD}}}$, due to the cavity, we use the master equation to obtain a system of equations for the mean values of operators $\langle\sigma _{{\mathrm{DD}}}\rangle$, $\langle a\sigma _{{\mathrm{gD}}}^\dagger\rangle$, and $\langle\sigma _{{\mathrm{gD}}}^\dagger \sigma _{{\mathrm{gB}}}\rangle$, and apply the adiabatic approximation to obtain the steady states (see Supplementary Note).

We also decompose the emission spectra into the bright- and dark-state contributions. To that end, we approximate the time evolution of the plasmon annihilation operator $a$ in the adiabatic approximation and express the emission spectrum in terms of the excitonic operators. In particular, we assume that we can split the total photoluminescence spectrum, $S_{{\mathrm{em}}}\left( \omega \right) = S_{{\mathrm{em}}\left( {\mathrm{B}} \right)}\left( \omega \right) + S_{{\mathrm{em}}\left( {\mathrm{D}} \right)}(\omega )$, into the contributions that emerge due to the bright exciton, $S_{{\mathrm{em}}\left( {\mathrm{B}} \right)}(\omega )$, and due to the dark exciton, $S_{{\mathrm{em}}\left( {\mathrm{D}} \right)}(\omega )$, as displayed in Fig. 5b. The details of the PL calculation for each of the contributions can be found in the Supplementary Note.

## Supplementary information

Supplementary Information

## Data Availability

The datasets generated during and/or analysed during the current study are available from the corresponding author upon reasonable request.

## Code availability

Codes used to generate simulated data are available from the corresponding author upon request.
