# Supplementary material for: Complex plasmon-exciton dynamics revealed through quantum dot light emission in a nanocavity
Source: Nat Commun. 2021 Feb 26;12:1310. doi: 10.1038/s41467-021-21539-z (PMC7910578; doi:10.1038/s41467-021-21539-z)
Supplement: Supplementary file 1 — Supplementary Information [file 41467_2021_21539_MOESM1_ESM.pdf]

Supplementary Information

Complex plasmon-exciton dynamics revealed through quantum dot light emission in a nanocavity

Satyendra Nath Gupta[1,&], Ora Bitton[2,&], Tomas Neuman[3,4,&], Ruben Esteban[3,4], Lev Chuntonov[5], Javier Aizpurua[3,4,*], and Gilad Haran[1,*]

[1]Department of Chemical and Biological Physics, Weizmann Institute of Science, POB 26, Rehovot 7610001, Israel,

[2]Department of Chemical Research Support, Weizmann Institute of Science, POB 26, Rehovot 7610001, Israel,

[3]Materials Physics Center CSIC-UPV/EHU, Paseo Manuel de Lardizabal 5, 20018 Donostia-San Sebastián, Spain

[4]Donostia International Physics Center DIPC, Paseo Manuel de Lardizabal 4, 20018 Donostia-San Sebastián, Spain

[5]Schulich Faculty of Chemistry, Technion-Israel Institute of Technology, Haifa, Israel.

*Correspondence: gilad.haran@weizmann.ac.il, aizpurua@ehu.eus

[&]These authors contributed equally to the work.

Supplementary Figures

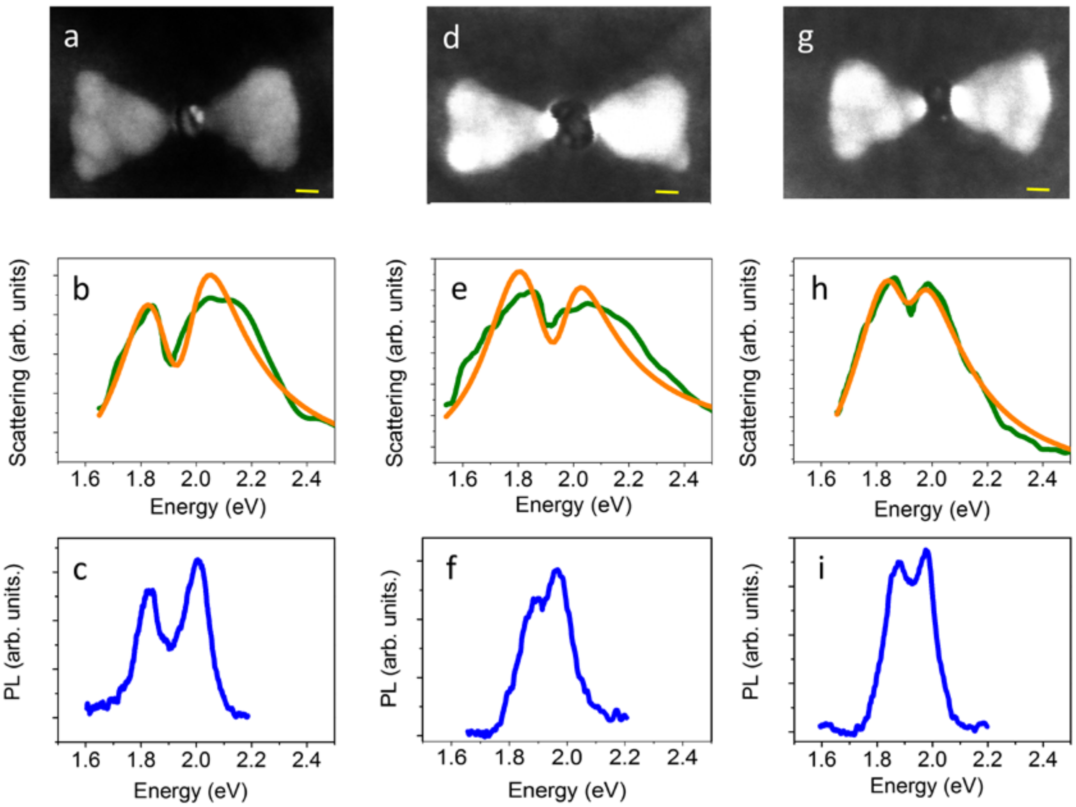

Supplementary Figure 1: Additional examples of the spectroscopy of devices loaded with QDs. STEM images (panels a,d,g), dark-field scattering spectra (panels b,e,h) and PL spectra (panels c,f,i) of three bowties containing QDs. The orange lines in panels b,e & h are fits to the coupled-oscillator model described in the legend of Supplementary Figure 2. The obtained coupling strengths are 81.3±0.6, 77.2±1.0 and 54.4±0.5 meV, respectively. Scale bars in panels a,d,g represent 20 nm.

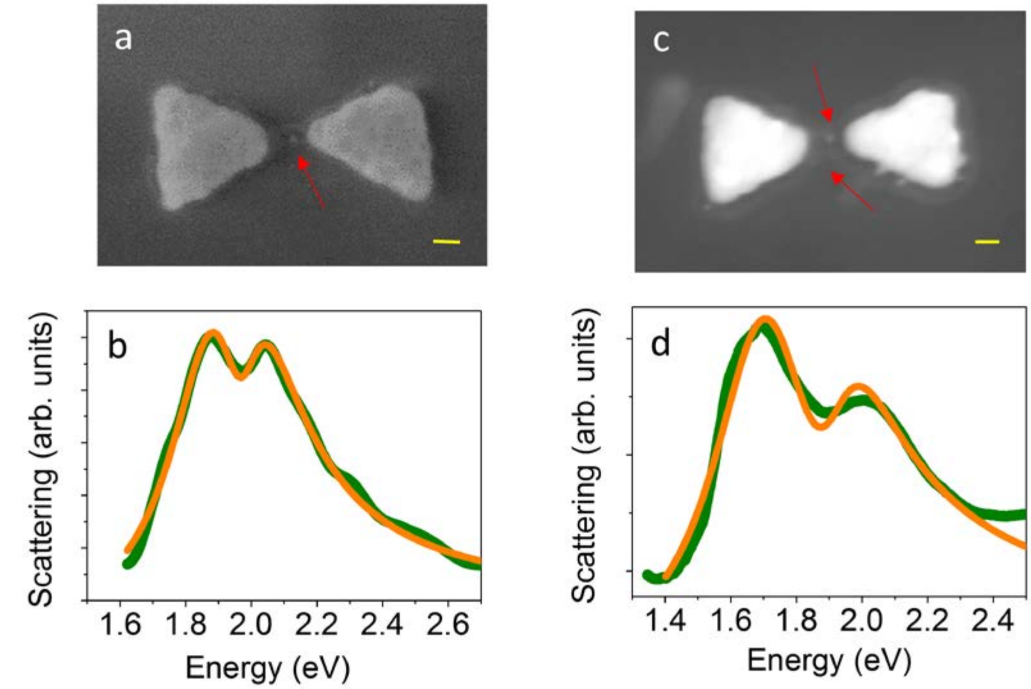

Supplementary Figure 2: Coupled-oscillator model fits of scattering spectra: STEM images (panels a&c) and dark-field scattering spectra (panels b&d) of two bowties containing QDs shown in Figure 1 of the main text. The orange lines in panel b & d are fits to the coupled-oscillator model [27,28]:

$$S(\omega) \propto \omega^4 \left| \frac{(\omega_e^2 - \omega^2 - i\gamma_e \omega)}{(\omega^2 - \omega_p^2 + i\gamma_p \omega)(\omega^2 - \omega_e^2 + i\gamma_e \omega) - 4\omega^2 g^2} \right|^2,$$

where ω_e and γ_e are the emitter resonance frequency and decay rate respectively, ω_p and γ_p are the plasmon frequency and plasmon decay rate, respectively and g is the coupling rate. The obtained values of g are 52.6±0.3 and 103.5±1.1 meV for panels b and d, respectively. In these fits, as well as the fits in Supplementary Figure 1, we fixed the values of γ_e (132 meV) and γ_p (395 meV), based on the measured line widths of the individual QD PL and scattering spectra of the empty bowtie. A baseline parameter was used in the fitting in order to take care of a constant background signal.

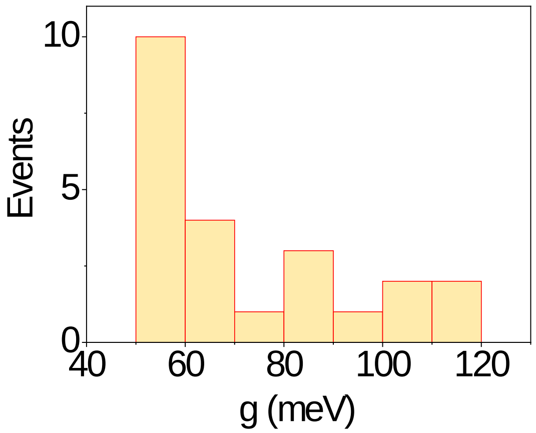

Supplementary Figure 3: Coupling rate values. Histogram shows the distribution of the values of coupling rates, g, obtained from fits of the scattering spectra using the coupled-oscillator model.

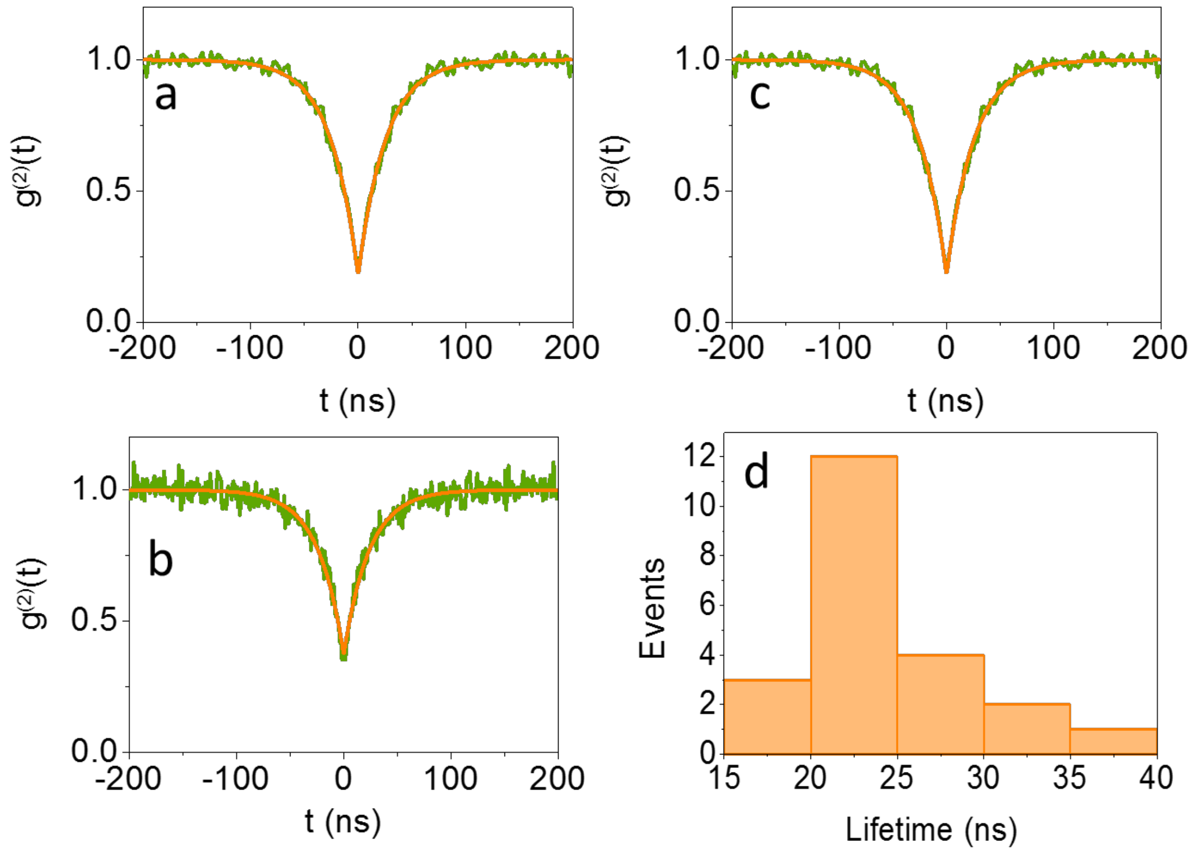

Supplementary Figure 4: Second-order photon correlation function of QDs on glass. (a-c) Three additional examples of measured correlation function of individual QDs on a glass substrate. (d) Distribution of the lifetimes of the excitons of 22 QDs on glass, obtained from analysis of $g^{(2)}$(t) functions.

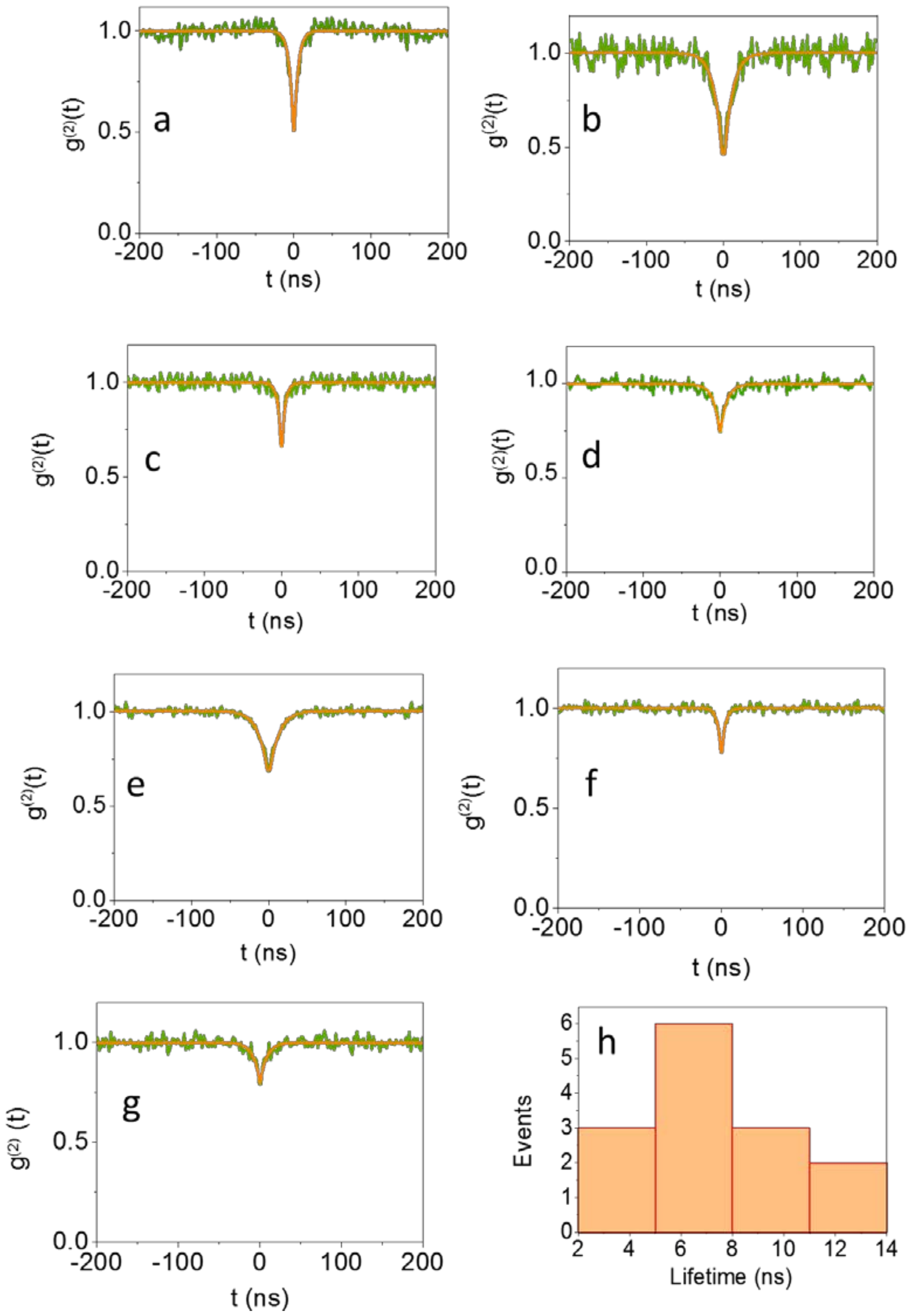

Supplementary Figure 5: Second-order photon correlation function of QDs coupled to a PC. (a-g) Additional examples of correlation functions measured from strongly coupled QDs. (h) Distribution of the lifetimes of 14 coupled plasmonic cavity-QD systems, obtained from analysis of $g^{(2)}$(t) functions.

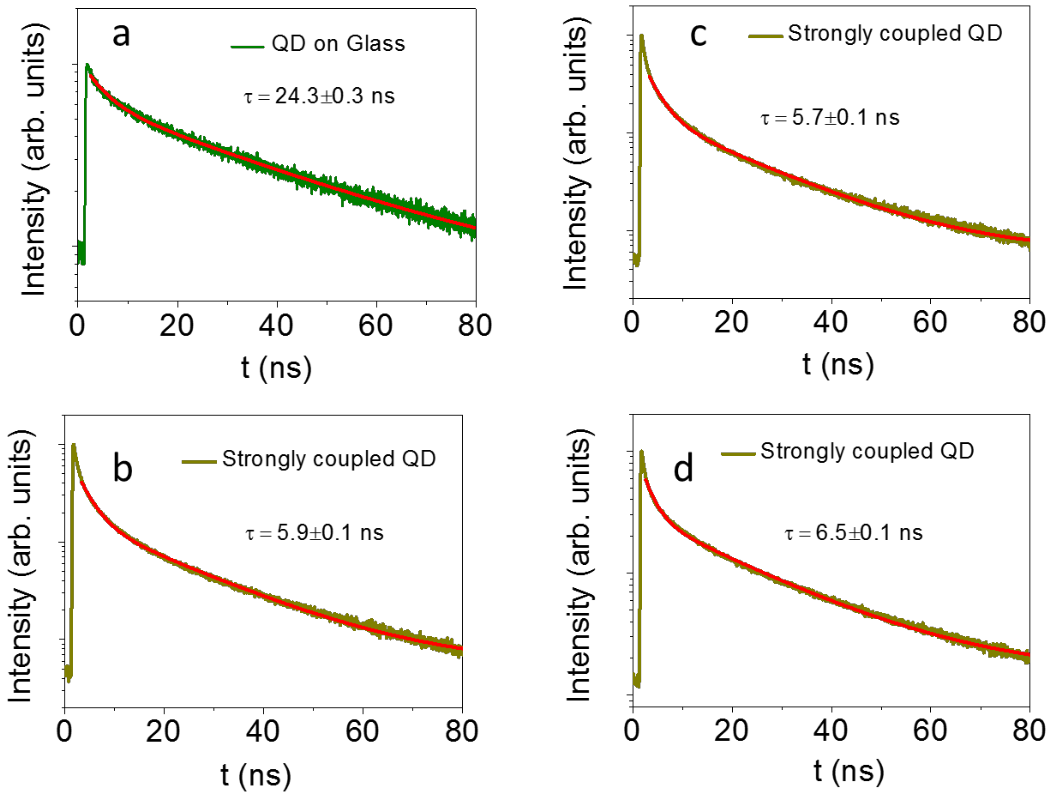

Supplementary Figure 6: Time-resolved PL measurements of QDs. (a) Measurement of QDs on a glass substrate. (b-d) Measurements of QDs coupled to PCs. The curves show the total decay of fluorescence, due to both radiative and non-radiative effects. The solid red lines are bi-exponential fits to the experimental data. The average lifetime (i.e. the weighted average of the two lifetimes obtained from the fits) is given in each panel.

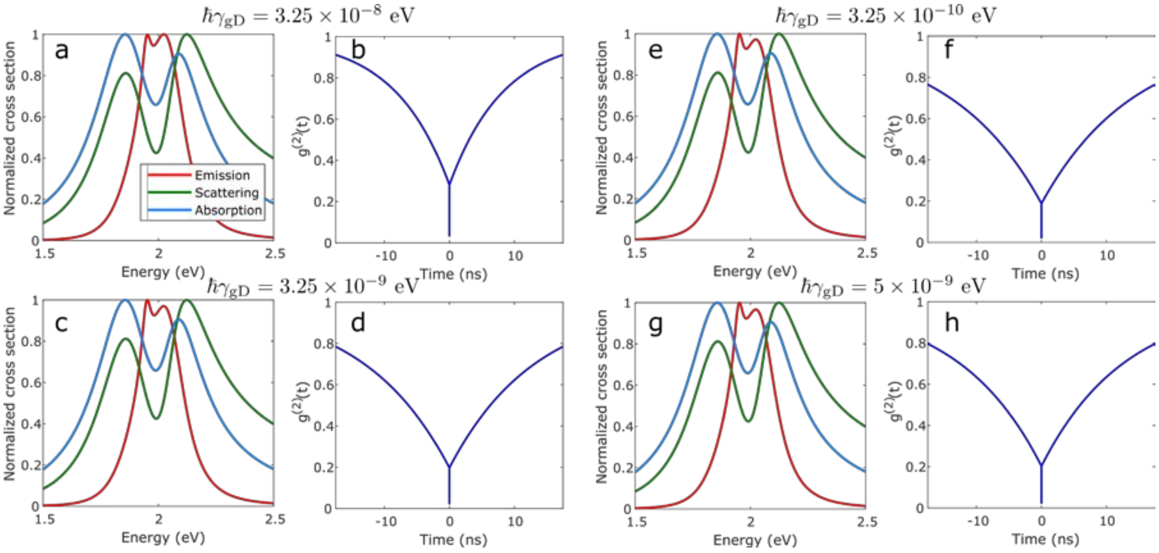

Supplementary Figure 7: Dependence of spectra and correlation functions on the intrinsic decay rate of the dark exciton γ_{gD}. A set of calculations of the emission, scattering and absorption of the antenna-emitter hybrid together with the correlation function of the emission, $g^{(2)}$, for situations in which the intrinsic decay rate of the dark exciton, γ_{gD}, is modified. The value of the dark exciton decay rate used is $\hbar\gamma_{gD}$= 3.25x10^{-8} eV (a,b); $\hbar\gamma_{gD}$= 3.25x10^{-9} eV (c,d); $\hbar\gamma_{gD}$= 3.25x10^{-10} eV (e,f); and $\hbar\gamma_{gD}$= 5x10^{-9} eV (g,h). The main features of the spectra and emission statistics are very robust against this parameter. This is due to the fact that the dynamics are governed by the rate of energy transfer between the dark and the bright exciton rather that the intrinsic dark exciton lifetime itself.

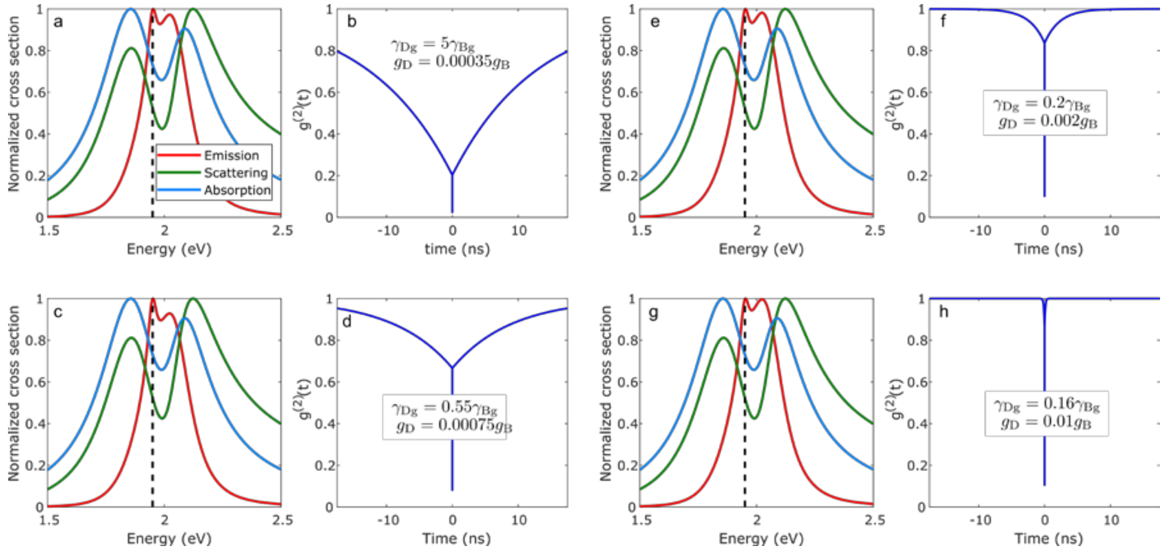

Supplementary Figure 8: Influence of the model parameters on simulated spectra and correlation functions. Model parameters are modified here in order to test how they affect predictions for experimental observables. (a,c,e,g) Emission (red line), absorption (blue line), and scattering (green line) spectra and (b,d,f,h) $g^{(2)}$(t) functions calculated from the theoretical model considering different ratios of incoherent pumping ($\gamma_{\mathrm{Bg}}/\gamma_{\mathrm{Dg}}$, with $\hbar\gamma_{\mathrm{Bg}} =$ 1 neV in all cases) and different values of the Jaynes-Cummings coupling constant of the dark exciton with plasmons, g_{D}. The values are given in the inset and apply to each pair [(a,b), (c,d), (e,f), and (g,h)] separately. All other parameters are listed in Table 1 of the main text. The spectral response is almost identical in all cases, but the form of $g^{(2)}$(t) very sensitively depends on both $\gamma_{\mathrm{Bg}}/\gamma_{\mathrm{Dg}}$ and g_D. The ratio $\gamma_{\mathrm{Bg}}/\gamma_{\mathrm{Dg}}$ controls the relative contribution of the fast component of the decay with respect to the slow one. On the other hand, large values of g_{D} give rise to a Purcell effect that shortens the lifetime of the dark exciton, thus shortening the lifetime associated with the slow decay. By fitting an exponential function of the form $f(t) = 1 - Ae^{-\frac{t}{T}}$ to the slow-decaying tails of the correlation function $g^{(2)}$(t) we have obtained the following lifetimes of the dark exciton: (b) T=12.8 ns, (d) T=8.9 ns, (f) T=2.3 ns, (h) T=0.1 ns.

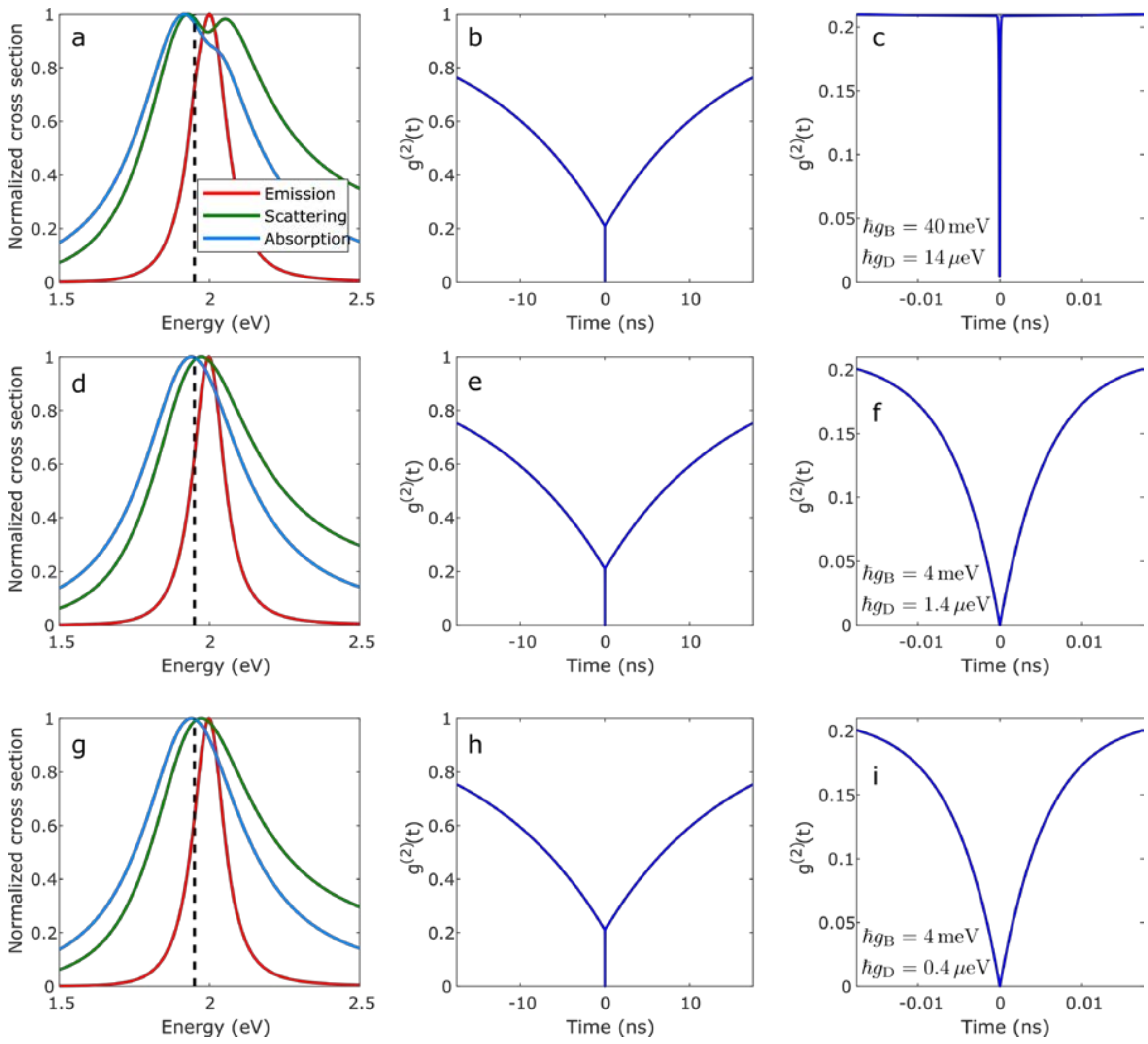

Supplementary Figure 9: Influence of the plasmon-exciton couplings on spectra and on the time resolution of the fast component in photon correlation functions. Optical properties of emission, scattering, and absorption (a,d,g) of the antenna-emitter hybrid for different parameters of the bright and dark exciton coupling to the antenna, g_B and g_D, respectively. The second-order correlation function of the emission $g^{(2)}$ for the same coupling strengths are displayed in (b,e,h). A zoom-in of the correlation function in a shorter time scale is provided in (c,f,i). The coupling parameters considered are $\hbar g_B$= 40 meV; $\hbar g_D$= 14 µeV in (a,b,c); $\hbar g_B$= 4 meV; $\hbar g_D$= 1.4 µeV in (d,e,f); and $\hbar g_B$= 4 meV; $\hbar g_D$= 0.4 µeV in (g,h,i). One can observe that when the coupling of the bright state with the plasmon is decreased to 4 meV (f,i), the fast component of the biexponential decay becomes slower, giving rise to a broader dip in the correlation function, of the order of approximately 10 picoseconds, which might bring the observation of this fast-decaying component to the edge of experimental

observation. However, a coupling of 4 meV is in the weak coupling regime, and therefore no fingerprint of strong coupling would be observable any more under these conditions.

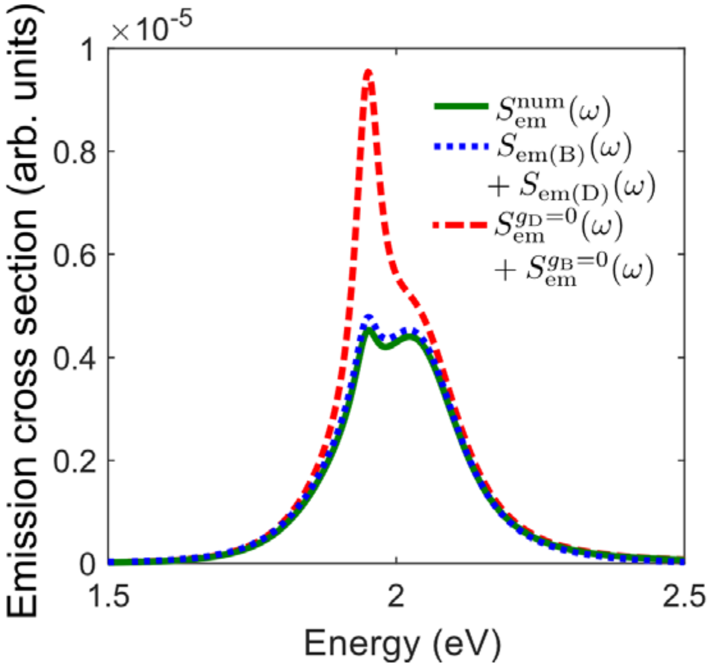

Supplementary Figure 10: Comparison of the PL spectrum obtained from the numerical calculation and from the analytical decomposition. The result of a full numerical calculation of the emission spectra (green line) is compared with the analytical result as in Fig. 5(b) of the main text (blue dotted line), and with the sum of numerically calculated spectra $S_{\rm em}^{g_{\rm D}=0}(\omega) + S_{\rm em}^{g_{\rm B}=0}(\omega)$ (red dashed line), as in Fig. 5(a) of the main text. All spectra are calculated for the set of parameters specified in Table 1 of the main text.

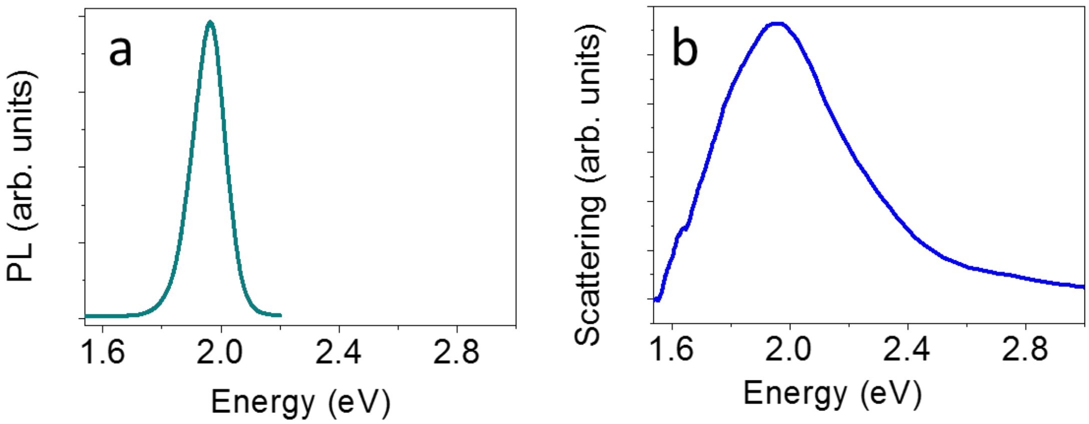

Supplementary Figure 11: Spectroscopy of a bare QD and an empty plasmonic cavity. (a) PL spectrum of a bare QD on a glass substrate. (b) Dark-field scattering spectrum of an empty plasmonic bowtie.

Supplementary Tables

Supplementary Table 1: Decay times and amplitudes extracted from biexponential fits to time-resolved PL curves.

	a_1	τ_1 (ns)	a_2	τ_2 (ns)	$\langle\tau\rangle$ (ns)
**QD on glass**	0.43	4.33	0.57	39.16	24.18
**Coupled QD 1**	0.82	2.91	0.18	19.09	5.82
**Coupled QD 2**	0.82	2.89	0.18	18.51	5.70
**Coupled QD 3**	0.75	2.30	0.25	19.55	6.61

a_1 and a_2 are amplitudes and τ_1 and τ_2 are decay times, while $\langle\boldsymbol{\tau}\rangle$ is the average decay time.

Supplementary Note: Analytical treatment of the plasmon-exciton dynamics and photoluminescence.

To obtain an analytical insight into the exciton dynamics and the origin of the features appearing in the excitonic light-emission spectra, we use the separation of time scales that naturally emerges in the system under study. The bright exciton is coupled to the single mode of the plasmonic structure with a coupling strength g_{B} comparable to the intrinsic losses κ of the plasmon, and therefore the dynamics of the plasmon and the bright exciton must be treated on the same footing. The dark exciton, however, is coupled to the plasmon with a coupling strength $g_{\mathrm{D}} \ll g_{\mathrm{B}}$, which allows us to treat this coupling perturbatively. This separation of time scales allows us to obtain an effective cavity-induced decay rate of the dark exciton $\gamma_{\mathrm{Pur}}^{\mathrm{D}}$ (Purcell effect) and the resulting dark-exciton steady-state population. Additional incoherent coupling between the dark exciton and the bright exciton with rates γ_{BD} (dark-to-bright-exciton incoherent coupling rate) and γ_{DB} (bright-to-dark-exciton incoherent coupling rate) are also included in the model. As we demonstrate below, their role is mainly to influence the relative distribution of excitonic population between the bright and the dark exciton and thus rescale the amplitudes of the features in the emission spectra arising from the bright- and the dark-exciton emission, respectively.

Effective decay rate of the dark exciton

In this section we provide details on the calculation of the effective dynamics (decay) of the dark exciton.

We calculate the effective decay rate of the dark exciton interacting with the cavity using the master equation. We first obtain approximate expressions for the following operator mean values:

$$\frac{d}{dt}\langle\sigma_{\mathrm{DD}}\rangle = -(\gamma_{\mathrm{gD}} + \gamma_{\mathrm{BD}})\langle\sigma_{\mathrm{DD}}\rangle - ig_{\mathrm{D}}\left\langle a\sigma_{\mathrm{gD}}^{\dagger}\right\rangle + ig_{\mathrm{D}}\langle a^{\dagger}\sigma_{\mathrm{gD}}\rangle + \gamma_{\mathrm{Dg}}, \qquad (1)$$

$$0 \approx \frac{d}{dt}\left\langle a\sigma_{\mathrm{gD}}^{\dagger}\right\rangle = \left[-i(\omega_{\mathrm{pl}} - \omega_{\mathrm{D}}) - \left(\frac{\Gamma_{\mathrm{D}}}{2} + \frac{\kappa}{2}\right)\right]\left\langle a\sigma_{\mathrm{gD}}^{\dagger}\right\rangle - ig_{\mathrm{D}}\langle\sigma_{\mathrm{DD}}\rangle - ig_{\mathrm{B}}\left\langle\sigma_{\mathrm{gD}}^{\dagger}\sigma_{\mathrm{gB}}\right\rangle, \qquad (2)$$

$$0 \approx \frac{d}{dt}\left\langle \sigma_{\mathrm{gD}}^{\dagger}\sigma_{\mathrm{gB}}\right\rangle = \left[-i(\omega_{\mathrm{B}}-\omega_{\mathrm{D}}) - \left(\frac{\Gamma_{\mathrm{D}}}{2}+\frac{\Gamma_{\mathrm{B}}}{2}\right)\right]\left\langle \sigma_{\mathrm{gD}}^{\dagger}\sigma_{\mathrm{gB}}\right\rangle - i g_{\mathrm{B}}\left\langle a\sigma_{\mathrm{gD}}^{\dagger}\right\rangle, \tag{3}$$

with $\sigma_{\mathrm{gB}} = |\mathrm{g}\rangle\langle \mathrm{e_B}|$, $\sigma_{\mathrm{gD}} = |\mathrm{g}\rangle\langle \mathrm{e_D}|$ and $\sigma_{\mathrm{DD}} = |\mathrm{e_D}\rangle\langle \mathrm{e_D}|$. On the right-hand side of Supplementary Eq. (2) and (3) we have neglected $g_{\mathrm{D}}\langle a^{\dagger}\sigma_{\mathrm{gB}}\rangle \approx 0$ and $g_{\mathrm{D}}\langle a^{\dagger}a\rangle \approx 0$, and used the adiabatic approximation to set the time derivatives in Supplementary Eqs. (2) and (3) equal to zero. From Supplementary Eqs. (2-3) we obtain:

$$\left\langle a\sigma_{\mathrm{gD}}^{\dagger}\right\rangle \approx \frac{i g_{\mathrm{D}}\left[\frac{(\gamma_{\mathrm{B}}+\Gamma_{\mathrm{D}})}{2} + i(\omega_{\mathrm{B}}-\omega_{\mathrm{D}})\right]\langle \sigma_{\mathrm{DD}}\rangle}{g_{\mathrm{B}}^{2} + \left[\frac{(\gamma_{\mathrm{B}}+\Gamma_{\mathrm{D}})}{2} + i(\omega_{\mathrm{B}}-\omega_{\mathrm{D}})\right]\left[\frac{\kappa}{2} + i(\omega_{\mathrm{pl}}-\omega_{\mathrm{D}})\right]}. \tag{4}$$

Supplementary Eqs. (1-3) also yield an effective equation of motion for $\langle \sigma_{\mathrm{DD}}\rangle$ upon insertion of the approximate steady-state solution for $\left\langle a\sigma_{\mathrm{gD}}^{\dagger}\right\rangle$ and $\langle a^{\dagger}\sigma_{\mathrm{gD}}\rangle = \left\langle a\sigma_{\mathrm{gD}}^{\dagger}\right\rangle^{*}$:

$$\frac{d}{dt}\langle \sigma_{\mathrm{DD}}\rangle \approx -(\gamma_{\mathrm{gD}} + \gamma_{\mathrm{BD}} + \gamma_{\mathrm{Pur}}^{\mathrm{D}})\langle \sigma_{\mathrm{DD}}\rangle + \gamma_{\mathrm{Dg}},$$

where

$$\gamma_{\mathrm{Pur}}^{\mathrm{D}} \approx \frac{g_{\mathrm{D}}^{2}\left[g_{\mathrm{B}}^{2}(\Gamma_{\mathrm{B}}+\Gamma_{\mathrm{D}}) + (\kappa+\Gamma_{\mathrm{D}})\left(\frac{(\Gamma_{\mathrm{B}}+\Gamma_{\mathrm{D}})^{2}}{4} + (\omega_{\mathrm{B}}-\omega_{\mathrm{D}})^{2}\right)\right]}{g_{\mathrm{B}}^{4} + 2g_{\mathrm{B}}^{2}D_{1} + D_{2}}, \tag{5}$$

and

$$D_{1} = \left[\frac{(\kappa+\Gamma_{\mathrm{D}})(\Gamma_{\mathrm{B}}+\Gamma_{\mathrm{D}})}{4} - (\omega_{\mathrm{pl}}-\omega_{\mathrm{D}})(\omega_{\mathrm{B}}-\omega_{\mathrm{D}})\right],$$

$$D_{2} = \left[\left(\omega_{\mathrm{pl}}-\omega_{\mathrm{B}}\right)^{2} + \frac{(\kappa+\Gamma_{\mathrm{D}})^{2}}{4}\right]\left[[(\omega_{\mathrm{D}}-\omega_{\mathrm{B}})^{2} + \frac{(\Gamma_{\mathrm{B}}+\Gamma_{\mathrm{D}})^{2}}{4}\right].$$

With the effective decay rate in hand, we can obtain the steady-state populations of the dark exciton, $\langle \sigma_{\mathrm{DD}}\rangle$:

$$\langle \sigma_{\mathrm{DD}}\rangle \approx \frac{\gamma_{\mathrm{Dg}}}{\Gamma_{n_{\mathrm{D}}}}, \tag{6}$$

Where $\Gamma_{n_{\mathrm{D}}} = \gamma_{\mathrm{gD}} + \gamma_{\mathrm{BD}} + \gamma_{\mathrm{Pur}}^{\mathrm{D}}$ and we have assumed that the population of the bright state $\langle\sigma_{\mathrm{BB}}\rangle$ is small so that $\gamma_{\mathrm{gD}} \gg \gamma_{\mathrm{DB}}\langle\sigma_{\mathrm{BB}}\rangle$ (with $\sigma_{\mathrm{BB}} = |\mathrm{e_B}\rangle\langle\mathrm{e_B}|$).

Decomposition of the emission spectra into the bright- and dark-state contribution

Next, we calculate the approximate photoluminescence spectrum of the light emitted from the plasmonic cavity and directly link the light emission to the underlying excitonic dynamics. To that end, we approximate the time evolution of the plasmon annihilation operator a in the adiabatic approximation and express the emission spectrum in terms of the excitonic operators. In particular, we assume that we can split the total photoluminescence spectrum, $S_{\mathrm{em}}(\omega) = S_{\mathrm{em(B)}}(\omega) + S_{\mathrm{em(D)}}(\omega)$, into the contributions that emerge due to the bright exciton, $S_{\mathrm{em(B)}}(\omega)$, and dark exciton, $S_{\mathrm{em(D)}}(\omega)$, respectively:

$$S_{\mathrm{em(B)}}(\omega) = 2\omega^4 Re\left\{\int_0^\infty \langle\, a_{\mathrm{B}}^\dagger(0)a_{\mathrm{B}}(t)\rangle\, e^{i\omega t}dt\,\right\}, \tag{7}$$

$$S_{\mathrm{em(D)}}(\omega) = 2\omega^4 Re\left\{\int_0^\infty \langle\, a_{\mathrm{D}}^\dagger(0)a_{\mathrm{D}}(t)\rangle\, e^{i\omega t}dt\,\right\}. \tag{8}$$

Here we have used the lower index B and D to explicitly mark the dynamics of the bright and the dark exciton, respectively. For brevity we omit this index in the following discussion of the individual spectral contributions, unless it is needed for clarity.

Photoluminescence spectrum due to the bright exciton, $S_{\mathrm{em(B)}}(\omega)$

To obtain $S_{\mathrm{em(B)}}(\omega)$ we use the quantum regression theorem (QRT), assuming again that the dark exciton does not significantly influence the dynamics of the coupling between the plasmon and the bright exciton:

$$\frac{d}{dt}\langle a^\dagger(0)a(t)\rangle = \left(-i\omega_{\mathrm{pl}} - \frac{\kappa}{2}\right)\langle a^\dagger(0)a(t)\rangle - ig_{\mathrm{B}}\langle a^\dagger(0)\sigma_{\mathrm{gB}}(t)\rangle,$$

$$\frac{d}{dt}\langle a^\dagger(0)\sigma_{\mathrm{gB}}(t)\rangle = \left(-i\omega_{\mathrm{B}} - \frac{\Gamma_{\mathrm{B}}}{2}\right)\langle a^\dagger(0)\sigma_{\mathrm{gB}}(t)\rangle - ig_{\mathrm{B}}\langle a^\dagger(0)a(t)\rangle.$$

After inserting the result into Supplementary Eq. (7) we obtain:

$$S_{\mathrm{em(B)}}(\omega) = 2\omega^4\, Re\left\{\frac{ig_{\mathrm{B}}\langle a^\dagger\sigma_{\mathrm{gB}}\rangle + i\left(\omega - \omega_{\mathrm{B}} + \frac{i\Gamma_{\mathrm{B}}}{2}\right)\langle a^\dagger a\rangle}{\left(\omega - \omega_{\mathrm{B}} + \frac{i\Gamma_{\mathrm{B}}}{2}\right)\left(\omega - \omega_{\mathrm{pl}} + \frac{i\kappa}{2}\right) - g_{\mathrm{B}}^2}\right\}, \tag{9}$$

with

$$\langle a^{\dagger}\sigma_{\mathrm{gB}}\rangle = \frac{g_{\mathrm{B}}\kappa[\omega_{\mathrm{B}}-\omega_{\mathrm{pl}}+i(\kappa+\Gamma_{\mathrm{B}})/2\ ]\big(\gamma_{\mathrm{Bg}}+\gamma_{\mathrm{BD}}\langle\sigma_{\mathrm{DD}}\rangle\big)}{g_{\mathrm{B}}^{2}(\Gamma_{\mathrm{B}}+\kappa)(\gamma_{\mathrm{B}}+\kappa)+\gamma_{\mathrm{B}}\kappa\left[\left(\omega_{\mathrm{B}}-\omega_{\mathrm{pl}}\right)^{2}+\frac{\kappa+\Gamma_{\mathrm{B}}}{4}\right]},$$

$$\langle a^{\dagger}a\rangle = \frac{g_{\mathrm{B}}^{2}(\Gamma_{\mathrm{B}}+\kappa)(\gamma_{\mathrm{Bg}}+\gamma_{\mathrm{BD}}\langle\sigma_{\mathrm{DD}}\rangle)}{g_{\mathrm{B}}^{2}(\Gamma_{\mathrm{B}}+\kappa)(\gamma_{\mathrm{B}}+\kappa)+\gamma_{\mathrm{B}}\kappa\left[\left(\omega_{\mathrm{B}}-\omega_{\mathrm{pl}}\right)^{2}+\frac{\kappa+\Gamma_{\mathrm{B}}}{4}\right]},$$

Where $\gamma_{\mathrm{B}} = \gamma_{\mathrm{gB}} + \gamma_{\mathrm{DB}}$ (i.e. it does not contain pure dephasing processes but only decay processes). The expressions for $\langle a^{\dagger}a\rangle$, $\langle a^{\dagger}\sigma_{\mathrm{gB}}\rangle$, $\langle\sigma_{\mathrm{BB}}\rangle$ were obtained from the following system of steady-state equations derived from the master equation:

$$\kappa\langle a^{\dagger}a\rangle + ig_{\mathrm{B}}\langle a^{\dagger}\sigma_{\mathrm{gB}}\rangle - ig_{\mathrm{B}}\left\langle a\sigma_{\mathrm{gB}}^{\dagger}\right\rangle = 0, \tag{10}$$

$$\gamma_{\mathrm{B}}\langle\sigma_{\mathrm{BB}}\rangle + ig_{\mathrm{B}}\left\langle a\sigma_{\mathrm{gB}}^{\dagger}\right\rangle - ig_{\mathrm{B}}\langle a^{\dagger}\sigma_{\mathrm{gB}}\rangle = \gamma_{\mathrm{Bg}} + \gamma_{\mathrm{BD}}\langle\sigma_{\mathrm{DD}}\rangle, \tag{11}$$

$$\left[i\left(\omega_{\mathrm{pl}}-\omega_{\mathrm{B}}\right)-\left(\frac{\Gamma_{\mathrm{B}}}{2}+\frac{\kappa}{2}\right)\right]\left\langle a\sigma_{\mathrm{gB}}^{\dagger}\right\rangle - ig_{\mathrm{B}}\big(\langle a^{\dagger}a\rangle-\langle\sigma_{\mathrm{BB}}\rangle\big) = 0, \tag{12}$$

$$\left[i\left(\omega_{\mathrm{B}}-\omega_{\mathrm{pl}}\right)-\left(\frac{\Gamma_{\mathrm{B}}}{2}+\frac{\kappa}{2}\right)\right]\langle a^{\dagger}\sigma_{\mathrm{gB}}\rangle + ig_{\mathrm{B}}\big(\langle a^{\dagger}a\rangle-\langle\sigma_{\mathrm{BB}}\rangle\big) = 0. \tag{13}$$

Emission spectrum due to the dark exciton $S_{em(D)}(\omega)$

The contribution to the emission spectrum arising due to the dark state can be obtained using the eigenvector perturbation theory to approximate the two-time correlation function $\langle a^{\dagger}(0)a(t)\rangle$. We can obtain from the QRT the following system of differential equations for the two-time correlation functions:

$$\frac{d}{dt}\begin{bmatrix}\langle a^\dagger(0)a(t)\rangle \\ \langle a^\dagger(0)\sigma_{\mathrm{gB}}(t)\rangle \\ \langle a^\dagger(0)\sigma_{\mathrm{gD}}(t)\rangle\end{bmatrix} =$$

$$\left(\underbrace{\begin{bmatrix}-i\omega_{\mathrm{pl}}-\frac{\kappa}{2} & -ig_{\mathrm{B}} & 0 \\ -ig_{\mathrm{B}} & -i\omega_{\mathrm{B}}-\frac{\Gamma_{\mathrm{B}}}{2} & 0 \\ 0 & 0 & -i\omega_{\mathrm{D}}-\frac{\Gamma_{\mathrm{D}}}{2}\end{bmatrix}}_{M_0} + \underbrace{\begin{bmatrix}0 & 0 & -ig_{\mathrm{D}} \\ 0 & 0 & 0 \\ -ig_{\mathrm{D}} & 0 & 0\end{bmatrix}}_{\delta M}\right)\begin{bmatrix}\langle a^\dagger(0)a(t)\rangle \\ \langle a^\dagger(0)\sigma_{\mathrm{gB}}(t)\rangle \\ \langle a^\dagger(0)\sigma_{\mathrm{gD}}(t)\rangle\end{bmatrix}, \qquad (14)$$

The first matrix in the parenthesis on the right-hand side, denoted as M_0, is responsible for the dynamics of the unperturbed system, whereas the second matrix in the parenthesis, denoted as δM, represents the perturbative coupling of the dark exciton to the strongly interacting system composed by the bright exciton and the plasmon. The differential equation can be formally solved using the eigenvalue decomposition of matrix $M = M_0 + \delta M$. If M has non-degenerate eigenvalues λ_i and corresponding left (right) eigenvectors y_i (x_i), the solution of the differential equation is:

$$A(t) = \sum_i c_i x_i e^{\lambda_i t},$$

with

$$A(t) = \begin{bmatrix}\langle a^\dagger(0)a(t)\rangle \\ \langle a^\dagger(0)\sigma_{\mathrm{gB}}(t)\rangle \\ \langle a^\dagger(0)\sigma_{\mathrm{gD}}(t)\rangle\end{bmatrix},$$

and the coefficients c_i are determined from the initial condition:

$$A(0) = \begin{bmatrix}\langle a^\dagger a\rangle \\ \langle a^\dagger\sigma_{\mathrm{gB}}\rangle \\ \langle a^\dagger\sigma_{\mathrm{gD}}\rangle\end{bmatrix},$$

as

$$\begin{bmatrix}c_1 \\ c_2 \\ c_3\end{bmatrix} = \begin{bmatrix}y_{1,1} & y_{1,2} & y_{1,3} \\ y_{2,1} & y_{2,2} & y_{2,3} \\ y_{3,1} & y_{3,2} & y_{3,3}\end{bmatrix}\begin{bmatrix}\langle a^\dagger a\rangle \\ \langle a^\dagger\sigma_{\mathrm{gB}}\rangle \\ \langle a^\dagger\sigma_{\mathrm{gD}}\rangle\end{bmatrix},$$

where $y_{i,j}$is the j-th component of the i-th left eigenvector. In the perturbative approach we first obtain the exact eigenvectors x_{0i} and y_{0i} of M_0. We further assume that the eigenvectors x_{01}, x_{02} (y_{01}, y_{02}) belong to the subspace describing the dynamics of the bright exciton interacting with the plasmon, and the vector x_{03} (y_{03}) belongs to the dark-exciton subspace of M_0. The approximate eigenvectors of M can be found as:

$$x_i \approx \sum_{ij} \epsilon_{ij} x_{0j},$$

where

$$\epsilon_{ij} = \frac{y_{0j}^{\mathrm{T}} \delta M x_{0i}}{(\lambda_{0i} - \lambda_{0j}) y_{0j}^{\mathrm{T}} x_{0j}}.$$

To obtain the approximate emission spectrum we further assume that the eigenvectors corresponding to the dynamics of the plasmon coupled with the bright exciton, x_1, x_2 (y_1, y_2), remain approximately unchanged (decoupled from the dark exciton):

$$x_1 \approx x_{01}, x_2 \approx x_{02},$$

but we apply perturbation theory to obtain the eigenvector x_3:

$$x_3 \approx \sum_i \epsilon_{3i} x_{0i}.$$

The solution then separates into two independent contributions that give rise to: (i) the emission from the bright exciton coupled with the plasmon, $\langle a_{\mathrm{B}}^{\dagger}(0) a_{\mathrm{B}}(t) \rangle = \sum_{i=1,2} c_i x_{i,1} e^{\lambda_i t}$(corresponding to the spectrum $S_{\mathrm{em(B)}}(\omega)$ shown above for$\lambda_i \approx \lambda_{0i}$, with the unperturbed eigenvalues λ_{0i} and where $x_{i,j}$is the j-th component of the i-th right eigenvector), and (ii) the emission due to the dark exciton, $\langle a_{\mathrm{D}}^{\dagger}(0) a_{\mathrm{D}}(t) \rangle = c_3 x_{3,1} e^{\lambda_3 t}$. After performing the algebraic manipulations and inserting the result into Supplementary Eq. (8) we obtain:

$$S_{\mathrm{em(D)}}(\omega) \approx 2\omega^4 \mathrm{Re} \left\{ \frac{B}{i(\omega_{\mathrm{D}} - \omega) + \frac{\Gamma_{\mathrm{D}}}{2}} \right\}, \quad (15)$$

with

$$B = \frac{ig_{\mathrm{D}}\left[\frac{(\gamma_{\mathrm{B}} - \Gamma_{\mathrm{D}})}{2} + i(\omega_{\mathrm{B}} - \omega_{\mathrm{D}})\right]\langle a^{\dagger}\sigma_{\mathrm{gD}}\rangle}{g_{\mathrm{B}}^2 + \left[\frac{(\gamma_{\mathrm{B}} - \Gamma_{\mathrm{D}})}{2} + i(\omega_{\mathrm{B}} - \omega_{\mathrm{D}})\right]\left[\frac{(\kappa - \Gamma_{\mathrm{D}})}{2} + i(\omega_{\mathrm{pl}} - \omega_{\mathrm{D}})\right]},$$

and

$$\langle a^{\dagger}\sigma_{\mathrm{gD}}\rangle \approx \frac{-ig_{\mathrm{D}}\left[\frac{(\gamma_{\mathrm{B}} + \Gamma_{\mathrm{D}})}{2} - i(\omega_{\mathrm{B}} - \omega_{\mathrm{D}})\right]\langle \sigma_{\mathrm{DD}}\rangle}{g_{\mathrm{B}}^2 + \left[\frac{(\gamma_{\mathrm{B}} + \Gamma_{\mathrm{D}})}{2} - i(\omega_{\mathrm{B}} - \omega_{\mathrm{D}})\right]\left[\frac{(\kappa + \Gamma_{\mathrm{D}})}{2} - i(\omega_{\mathrm{pl}} - \omega_{\mathrm{D}})\right]},$$

where $\langle \sigma_{\mathrm{DD}}\rangle = \langle |\mathrm{e_D}\rangle\langle \mathrm{e_D}|\rangle$.
